# Targeting Telomerase in Cancer: Vaccine-Based Strategies, Clinical Evidence, and Synergy with Immunotherapy

**DOI:** 10.3390/diseases14020080

**Published:** 2026-02-20

**Authors:** Stella Baliou, Manolis N. Tzatzarakis, Andreas G. Tsantes, Elena Vakonaki, Petros Ioannou, Michalis Kyriakakis, Eleftheria Hatzidaki, Iordanis Pelagiadis, Eftichia Stiakaki, Aristides Tsatsakis

**Affiliations:** 1 Laboratory of Toxicology, School of Medicine, University of Crete, 71003 Heraklion, Greece; 2 Laboratory of Haematology and Blood Bank Unit, “Attiko” Hospital, School of Medicine, National and Kapodistrian University of Athens, 12462 Athens, Greece; 3 Microbiology Department, “Saint Savvas” Oncology Hospital, 11522 Athens, Greece; 4 Department of Internal Medicine, School of Medicine, University of Crete, 71003 Heraklion, Greece; 5 Department of Neonatology/NICU, University Hospital of Heraklion, School of Medicine, University of Crete, 71003 Heraklion, Greece; 6 Department of Pediatric Hematology-Oncology and Autologous Hematopoietic Stem Cell Transplantation Unit, University Hospital of Heraklion and Laboratory of Blood Diseases and Childhood Cancer Biology, School of Medicine, University of Crete, 71003 Heraklion, Greece; 7 Universidad Ecotec, Km. 13.5 Samborondón, Samborondón EC092302, Ecuador; 8 Sechenov IM First State Medical University, Moscow 119991, Russia

**Keywords:** telomerase vaccines, immune checkpoint inhibitors (ICIs), clinical efficacy, molecular mechanisms

## Abstract

With each replication cycle, telomeres shorten. Telomerase can slow or reverse the rate of telomere shortening. In the era of cancer immunotherapy, telomerase is a promising tumor-associated antigen due to its widespread and specific expression in cancer cells and its strong immunogenicity. Interestingly, telomerase-based vaccines eradicate telomerase-expressing cancer cells by increasing antigen-specific T-cell responses rather than by directly inhibiting telomerase enzymatic activity as telomerase inhibitors function. To support this, telomerase-based vaccines, including DNA, mRNA, peptide-, and cell-based vaccines, have been evaluated in clinical settings to elucidate their molecular mechanisms of action. The aim of this review is to present the clinical effectiveness of telomerase vaccines alone or in combination with immunotherapy. In particular, the therapeutic effectiveness of telomerase vaccines is influenced by the tumor microenvironment and can be substantially increased by combining them with immune checkpoint inhibitors. To further optimize telomerase-based vaccines, we discuss translational challenges and highlight the need for further optimization.

## 1. Introduction

Telomeres are nucleoproteins that protect chromosome ends from damage caused by exonucleases, free radicals, and other harmful agents. They consist of shelterin complex proteins bound to repetitive nucleotide sequences [1]. In particular, telomeres consist of repeated nucleotide sequences (5′-TTAGGG-3′) and are embedded with a complex of six proteins called the shelterin complex: TRF1, TRF2, Rap1, TIN2, TPP1, and POT1. Telomeres prevent chromosomal termini from being misidentified as DNA double-strand breaks (DSBs), thereby hindering the inappropriate induction of the DNA-damage response (DDR) [2]. The shelterin protein complex functions to prevent telomeres from being recognized as DSBs. This inhibition of DDR ultimately sustains chromosomal integrity [3,4].

Each cell division reduces telomere length in eukaryotic cells through distinct mechanisms. First, the “end-replication problem” causes telomere shortening because DNA polymerase cannot duplicate the 3′ end of the lagging strand [5]. In particular, the region that the primer had previously filled at the 5′ end of each newly synthesized strand remains unoccupied since there is no free 3′–OH, taking into consideration that DNA replication is carried out by the enzyme DNA polymerase in the 5′ → 3′ direction [5]. Second, oxidative and DNA damage further accentuate telomere shortening [6]. Indeed, telomere shortening is accentuated through the detrimental effects of oxidative stress on DNA, as shown by the increased formation of 8-hydroxy-2-deoxyguanosine (8-oxodG) at telomeres [7,8,9,10]. Several lines of evidence support that telomere shortening is closely linked to cellular senescence, that increases the risk for age-related diseases. The phenotype of senescent cells is characterized by the persistent senescence-associated secretory phenotype (SASP), which promotes chronic inflammation and tissue dysfunction [4].

However, a reverse transcriptase called telomerase can circumvent the challenges mentioned above that cause telomere shortening [11]. Telomerase is a ribonucleoprotein enzyme complex that reduces the telomere shortening rate by introducing repeating nucleotide sequences to chromosomal ends [12]. Telomerase is composed of two main subunits: telomerase reverse transcriptase (TERT) and the telomerase RNA component (TERC), with associated proteins [6]. These associated proteins, which include dyskerin, NHP2, NOP10, and GAR1, support the structural stability of the telomerase ribonucleoprotein complex [6]. In a molecular setting, TERT is a catalytic reverse transcriptase subunit that requires its own RNA template to add short repetitive DNA sequences to chromosomal termini, thereby avoiding replicative senescence [6].

Under physiological conditions, telomerase is only weakly expressed in somatic tissues, but it is continuously expressed in developing sperm cells and embryonic stem cells to maintain genome integrity [13]. Telomerase can also be activated by certain adult cell types, including adult stem cells, especially in tissue-renewing contexts [14]. In this manner, telomerase activation may delay organismal aging, reduce telomere shortening linked to aging, and improve longevity and overall health span [15,16]. Conversely, telomerase is aberrantly reactivated in most cancers, rendering cancer cells uncontrolled proliferators through telomere length maintenance [13,17]. In this context, cancer cells achieve effective immortalization by maintaining their telomere length through telomerase overexpression, thereby inhibiting replicative senescence [18]. In more detail, telomerase activity is widely regarded as a crucial component of the longevity and replicative nature of cancer cells and is observed in 85–90% of human malignancies, ranging from primary tumor initiation to metastatic progression [11,19]. In particular, the overexpression of TERT appears to play an oncogenic role, leading to increased mitochondrial activity and activation of DDR and WNT/β-catenin signaling [20,21,22]. It was recently shown that TERT controls MYC-driven carcinogenesis irrespective of telomerase action [23]. For example, point mutations in the TERT promoter region are linked to increased telomerase expression in cancer [24]. Another study showed that genomic abnormalities in cutaneous melanoma (80%) can arise from mutations in the hTERT promoter region, leading to elevated hTERT expression [25]. Promoter mutations in the hTERT gene have been associated with increased TERT transcription, leading to upregulated telomerase activity [26]. In the other example, the increased recruitment of transcription factors belonging to the ETS (E26 transformation-specific) family at the promoter region of TERT has been shown to upregulate the MAPK pathway, which is necessary for increasing TERT expression [24]. Aside from its primary canonical role in maintaining telomere length, telomerase also participates in various non-canonical activities, such as epithelial–mesenchymal transitions (EMT), which are closely linked to the uncontrolled growth and progression of cancer cells [27]. As a result, telomerase activation is considered a hallmark of cancer [28].

Nevertheless, a smaller subset of malignancies maintains telomere length via the alternative lengthening of telomeres (ALT) pathway, ensuring the immortalization of cancer cells even when hTERT is not expressed [29]. The ALT mechanism operates in a subset of neoplasms (5–15%) that lack telomerase activity; these are primarily sarcomas and gliomas. This process relies on homologous recombination-mediated DNA synthesis to generate telomeric ends [28,30].

For this reason, hTERT has become the focus of multiple clinical trials examining its suitability as a cancer therapy in combination with immunotherapy, considering the prevalent expression of telomerase in malignancies and its significant function in tumor progression. In this regard, telomerase vaccines elicit the immune-mediated elimination of telomerase-expressing tumor cells instead of interfering with telomerase activity [31].

## 2. The Significance of Tumor Vaccines in Immunotherapy

Cancer cells sustain telomere maintenance through telomerase reactivation, enabling replicative immortality [32,33,34], rendering telomerase a “universal” cancer target. Telomerase-based therapies are categorized into telomerase inhibitors and telomerase vaccines, which have distinct mechanisms of action. On one hand, the mechanism of action of telomerase inhibitors involves interfering with telomerase activity, leading to telomere shortening and ultimately, tumor growth arrest or cell death [35]. When telomerase inhibitors are administered, cancer cells undergo telomere degradation due to the inhibition of the human telomerase RNA component (TR) or telomere uncapping due to the inhibition of human telomerase reverse transcriptase (hTERT) [36]. Nevertheless, identifying telomerase inhibitors has proven challenging, and there are currently no clinically approved methods that exploit this cancer target. The clinical efficacy of telomerase inhibitors is limited by their delayed therapeutic onset, the multiple required cell divisions to reach critically short telomeres, and toxicity in regenerating tissues. Since telomerase inhibitors rely on telomere attrition with each cell cycle, their anticancer effect requires prolonged treatment, suggesting that they do not immediately shorten telomeres enough to cause cell death, leading to a cellular “lag phase” in which resistant cancer clones can arise and proliferate [37]. Apart from this, the clinically approved telomerase inhibitor (imetelstat) has been used in hematologic malignancies, specifically myeloproliferative neoplasms, myelodysplastic syndromes, and AML [9,10], and has been reported to be associated with hematologic toxicities, e.g., thrombocytopenia/neutropenia [37,38]. On the other hand, telomerase vaccines exploit human reverse transcriptase (hTERT) as a tumor-associated antigen to induce an antigen-specific immune response [39]. Briefly, telomerase vaccines overcome telomere-shortening lag and provide long-lasting immune surveillance. In more detail, telomerase vaccines can generate telomerase-specific T lymphocytes capable of killing hTERT-expressing cancer cells immediately upon antigen presentation [37]. Compared to telomerase inhibitors that require repeated administration, telomerase vaccines can also induce immunologic memory and T cell clonal proliferation, contributing to persistent immune surveillance [31,40]. Indeed, the mechanism of telomerase-based vaccines is the immune-mediated eradication of telomerase-expressing tumor cells, creating long-term immunological memory by stimulating hTERT-specific CD4^+^ and/or CD8^+^ T cells [31,40]. Another advantage of the telomerase vaccine is that it not only targets hTERT peptides but also can eradicate tumor cells through the release of additional tumor antigens (epitope spreading) [31,40]. While telomerase vaccines demonstrate favorable safety profiles and consistent immunogenicity, their clinical efficacy remains context-dependent and is strongly influenced by tumor immune microenvironment features, underscoring the need for rational combination strategies rather than monotherapy.

There has been significant progress in immunotherapy, leading to the development of cancer vaccines. By targeting one or more cancer antigens, these vaccines aim to elicit an immune response against cancerous cells. Initially, William B. Coley discovered that several bacteria can selectively enter tumors and elicit immune responses [41,42]. In 1891, William B. Coley developed the first standardized cancer immunotherapy, which was used to treat malignant tumors. In particular, the “Coley Toxin,” made from streptococcal bacteria, was used in the treatment of patients with bone and soft-tissue sarcomas [43]. Since then, research on the TME in the development of vaccines has grown exponentially, resulting in favorable treatment outcomes for hepatitis B-related liver cancer, cervical cancer linked to melanoma, and colon and bladder cancer. In the 1950s, Ehrlich developed the theory of immunosurveillance, which states that the immune system suppresses the development of significant illness until immunity is weakened [44]. Based on this finding, T lymphocytes can recognize peptide antigens expressed on tumor cell surfaces in the presence of MHC molecules, providing a foundation for the resurgence of the concept of tumor immunosurveillance [45]. With this perspective, cancer vaccines emerged. These cutting-edge vaccines aim to target tumor antigens released in the TME, stimulating and strengthening the immune response against cancer cells. The main objective of cancer vaccines is to enhance the immune system’s ability to recognize and eradicate cancer cells while preventing tumor development, recurrence, and metastasis. Vaccines induce an immune response against specific cancer antigens. In a preclinical setting, the p540 and p865 TERT peptide approaches have been proven effective against prostate, breast, colon and lung cancer cell lines through increased recruitment of cytotoxic T lymphocytes [46].

In 1980, the first cancer vaccine was created using autologous tumor cells [47]. In the early 1990s, melanoma-associated antigen 1 was recognized as the first human tumor antigen, paving the way for the use of tumor antigens in cancer vaccines [48]. In 2010, prostate cancer was successfully treated using a dendritic cell-based vaccine (Sipuleucel-T) that led to prolonged survival but did not cure the cancer [49].

Early therapeutic cancer vaccines were unable to enhance de novo T cell responses because they targeted aberrantly produced tumor-associated antigens (TAAs) on tumor cells [50]. Tumor cells overexpress or exhibit abnormal expression of TAAs, which are normal biological proteins that circulate at low levels in healthy tissues. Since TAAs originate from self-proteins, the immune system may tolerate them, but they can still trigger autoimmune reactions. In addition, immune responses against TAAs are often weak, requiring adjuvants to enhance them.

Nowadays, therapeutic cancer neoantigen strategies are highly advantageous since they specifically target a novel antigen while avoiding peripheral and central tolerance, as well as the potential “off-target” tissue damage seen with earlier TAA-targeting approaches [51]. Antigens with a high mutational load typically facilitate the identification of neoantigens, increasing the tumor cell-specific immune response [52] due to intratumoral heterogeneity [53,54]. Neoantigens, also known as tumor-specific antigens, are non-self peptides that arise from somatic mutations, gene fusions, or viral oncogene activation in tumor cells [52,55]. They are highly tumor-specific because they are absent from normal cells. As a result, the immune system recognizes them as foreign, since there is no pre-existing immunological tolerance [52]. With this perspective, cancer vaccines were designed against specific neoantigens, thereby generating long-lasting and targeted anti-tumor immune responses against cancer cells using neoantigens that are well-tolerated in the human setting [56].

## 3. Types of Telomerase-Based Vaccines

Telomerase is usually inactive in most somatic cells but is activated in more than 85–90% of human cancers [13,17]. In several malignancies, the human telomerase reverse transcriptase (hTERT) is a key factor in cellular immortality [13,17]. Because of its nearly universal presence in tumors and limited expression in normal tissues, hTERT has become a tumor-associated antigen (TAA) and a promising target for cancer immunotherapy [46,57,58,59,60,61,62,63,64,65,66]. Given its ubiquitous expression in malignancies and its crucial role in tumor growth, hTERT has been the focus of several clinical trials evaluating its suitability as a universal vaccine antigen in cancer immunotherapy.

Several telomerase vaccine types, including peptide-based, DNA, mRNA, viral vector, and cell-based formulations, have been developed to target hTERT [39]. Examples of existing vaccines include peptide [short or synthetic long peptide (SLP)] vaccines; mRNA vaccines; cell vaccines, including dendritic-cell (DC) vaccines; and viral vector vaccines. The antigen epitope and HLA typing are important determinants for the development of peptide-based vaccines. Short peptide-based vaccines usually activate CD8^+^ T cytotoxic cells [39]. In contrast, SLP-based vaccines preferentially boost CD4^+^ Th1 immunity and allow cross-presentation to CD8^+^ T cells, leading to a widespread and long-lasting immune response [39]. In summary, peptide vaccines are often selected as the primary option for immunization because they are longer-lasting, safer, and easier to prepare than DNA and cell-based vaccines. The limitation of telomerase DNA vaccines is continuous antigen expression, and the drawback of dendritic vaccines is their standardization.

Although beyond the focus of this study, several alternative approaches, such as antisense oligonucleotides [23,67,68,69], viral therapy [70], small-molecule inhibitors [23,69,71,72], and enzyme inhibition [73], have been documented to target telomerase with beneficial effects against cancer.

### The Mechanisms of Action of Synthetic Long Peptide (SLP) Telomerase Vaccines

In the development of telomerase peptide vaccines, researchers first used short peptides (8–10 amino acids) that required prior patient HLA typing [74]. To stimulate both arms of cellular immunity, modern telomerase peptide vaccines employ synthetic long peptides (SLPs) (11–30 amino acids) that are present on both MHC class I and II molecules at the surface of APCs, activating CD8^+^ cytotoxic T cells and CD4^+^ helper T cells, thereby amplifying robust immune responses [75]. In this context, identifying a suitable telomerase TAA is the cornerstone of vaccine-based approaches, since telomerase is a target in more than 85% of malignancies [76]. The principle behind the action of SLP telomerase-based vaccines is to trigger both effective CD4^+^ and CD8^+^ T-cell immune responses. As a result, SLP-based telomerase vaccines tend to elicit broader, more sustained immune responses and are less constrained by HLA restriction than short peptide vaccines that induce HLA-restricted cytotoxic CD8^+^ T cell responses.

hTERT-derived long peptides are released by cancer cells during necrosis or apoptosis, and they are internalized, processed, and degraded by APCs into peptide fragments of varying lengths that bind to MHC class I or II molecules and are displayed on the APC surface. In particular, cytotoxic T lymphocytes and T helper cells are activated when antigenic peptide-MHC class I or II complexes are recognized by the T cell receptor (TCR) on the surface of CD8^+^ or CD4^+^ T cells, respectively [57,77]. As a result, SLP telomerase-based vaccines increase the likelihood of presentation across multiple HLA class II molecules and enable efficient cross-presentation on HLA class I, thereby supporting coordinated CD4^+^ and CD8^+^ T-cell activation.

**Figure 1:**
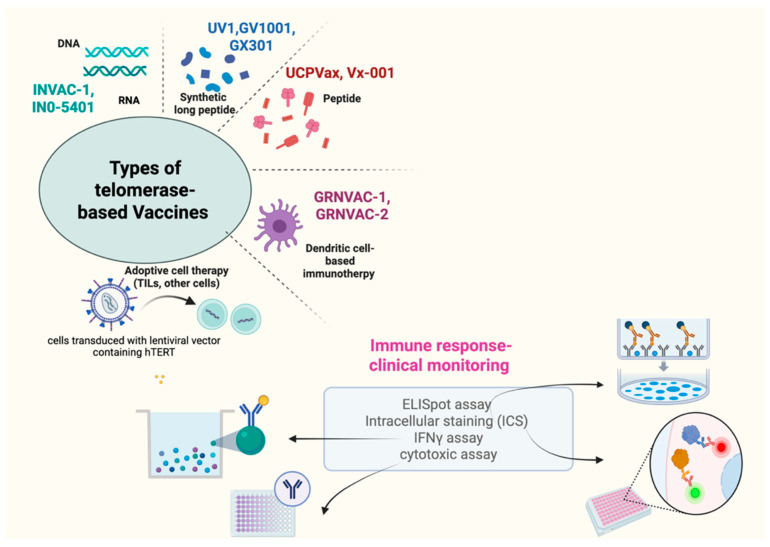
Telomerase vaccine types. (I) DNA telomerase vaccines in which plasmids containing unique epitopes of human telomerase reverse transcriptase (hTERT) DNA are typically electroporated to sensitize antigen-presenting cells (APCs), which then deliver antigens to stimulate T and B cells. (II) Synthetic long peptide (SLP) telomerase vaccines, in which antigen-presenting cells (APCs) are stimulated with multiple hTERT antigen epitopes, activating T and B cells. (III) Peptide telomerase vaccines. (IV) hTERT-targeting dendritic (DC) vaccines in which DCs are collected from patient blood, stimulated in vitro with hTERT antigen peptides, and then the vaccine is injected back into the patient to activate T and B cells. (V) In adoptive cell therapy, leukapheresis is used for the separation of leukocytes from peripheral blood and the in vitro activation of autologous lymphocytes is followed with telomerase antigen epitope. Then, the stimulated lymphocytes are reinjected into the patient, triggering the patient’s immune response. From an immunological perspective, the following immune assays have been used to evaluate the immunogenicity induced by telomerase-based vaccines and correlate it with the therapeutic efficiency of tumor vaccines. In particular, the enzyme-linked immunospot (ELISpot) assay, intracellular staining (ICS), interferon-gamma (IFNγ) assay, and cytotoxic assay have been used to address the immunological aspect of telomerase vaccines; Created in BioRender. Tsatsakis, A. (2025) https://BioRender.com/igpqbqm. (created with BioRender.com).

Some examples of SLP hTERT -based vaccines are UV1, GV1001, and GX301, which activate CD4^+^ and CD8^+^ Τ immune responses, regardless of HLA cross-presentation of hTERT antigen epitopes [78]. Some peptide vaccines have progressed to phase III clinical trials following preclinical testing and regulatory approval. Other telomerase peptide vaccines include Vx-001 and UCP-Vax (Figure 1). The SLP telomerase-based vaccines such as UV1, GV1001, and GX301 showed broader HLA coverage and more consistent immune responses, indicating that the vaccine format significantly influences immunogenicity. For example, UV1 elicits stronger immune responses than Vx-001 in NSCLC [79,80]. In fact, UV1 generates more robust immune responses than Vx-001 because it affects a broader range of HLA types.

The immunogenic properties of telomerase peptide vaccines are examined in greater detail. Initially, the UV1 vaccine comprises three elongated peptides (ranging from 15 to 30 amino acids) that overlap within the active catalytic region of hTERT. Importantly, these peptides are bound to MHC I and II complexes, which activate CD4^+^ and CD8^+^ T cell immune responses, respectively. Interestingly, the UV1 vaccine triggers strong CD4^+^ T helper responses, characterized by IFN-γ production and epitope spreading. The action of the UV1 vaccine increases T helper immune responses, which support the activation of CD8^+^ cytotoxic T cells and thereby contribute to memory responses. Additionally, the UV1 vaccine has been shown to prevent immune escape and demonstrate synergy with ICIs. Specifically, the UV1 vaccine targets epitopes in the hTERT catalytic core that are less prone to immune-escape mutations, thereby reducing the likelihood of tumor evasion and ensuring sustained antigen release. As a result, UV1 is more effective than Vx-001 at inducing clinically meaningful immune responses in NSCLC, which is linked to stronger and more persistent immune activation. In contrast to the above, the Vx-001 vaccine is based on a single, tailored cryptic peptide (TERT572Y) specific to HLA-A*0201. In particular, the Vx-001 telomerase vaccine elicits CD8^+^ cytotoxic T cell responses in 55–70% of patients that are often temporary and do not always correlate with improved survival [81]. The limitations of restricted epitope targeting are highlighted by the Vx-001 single-epitope vaccine, which frequently elicits short-lived CD8^+^ responses [81]. Even when immune responses are observed in NSCLC patients, the Vx-001 vaccine does not confer therapeutic benefit [81]. In this context, GX301 (multi-peptide) has also been proven to be more immunogenic than single-peptide vaccines. For example, the majority of prostate cancer (PC) patients have shown long-lasting immune activation with GX301 administration, but clinical response rates remained low, highlighting the need for biomarker-guided patient stratification [82]. Table 1 lists potential biomarkers that may be used to predict telomerase vaccines’ clinical efficacy and immune response.

Across telomerase-based vaccines, several biomarkers may be used to predict the vaccine response. To address this issue, immune monitoring has primarily relied on the accumulation of T-specific cells, cytokine patterns, epitope spreading, and TCR repertoire diversification, which are likely biomarkers associated with better outcomes due to immune system induction.

## 4. The Clinical Efficacy of Telomerase Peptide Vaccines

Numerous hTERT-targeting peptide vaccines have advanced to clinical testing. Characteristic examples include UV1 and GV1001, which elicit both CD4^+^ effector and CD8^+^ cytotoxic T-cell immune responses. By presenting hTERT-derived epitopes on both the MHC I and II pathways, these vaccines successfully trigger strong CD4^+^ helper T-cell and CD8^+^ cytotoxic T-cell activation. In specific cancer types, including melanoma, non-small-cell lung cancer, and pancreatic cancer, clinical studies have shown excellent safety profiles, strong immunogenicity, and indications of extended survival of telomerase peptide vaccines. Interestingly, the clinical efficacy of telomerase vaccines can be enhanced by combining them with immune checkpoint inhibitors (ICIs), as telomerase vaccines can expand T cells targeting telomerase and ICIs can counteract T-cell inhibitory signals, thereby attenuating T-cell exhaustion. The main concept behind combining telomerase vaccines with ICIs is that increased T-cell infiltration occurs when ICIs are ineffective.

Tumors with varying levels of telomerase (hTERT) expression or activity can have different patient outcomes and may respond differently to therapy. Among patients with colorectal cancer, those with high telomerase activity had a much worse prognosis than those with moderate or low telomerase activity [91]. However, higher telomerase levels do not reliably translate into better responses, as immune evasion mechanisms can affect the clinical efficacy of treatment [81]. In particular, high-telomerase tumors can be immune-evasive and unresponsive to telomerase vaccines, even when telomerase antigen expression is abundant. For example, TERT promoter mutations have been associated with higher tumor mutational burden, which, in turn, correlates with tumor immune infiltration and thus with response to therapies involving immune checkpoint inhibitors (ICIs) [92,93].

### 4.1. The Immune-Evasion Mechanisms of Immune Checkpoint Inhibitors (ICIs)

In general, malignancies with a poor prognosis have demonstrated clinical benefit from these ICIs, establishing ICIs as a cutting-edge option in immunotherapy [94]. However, a significant proportion of patients remain refractory to this form of immunotherapy [95].

Regarding the immunological checkpoint molecules involved in tumor immune evasion, the cytotoxic T-lymphocyte-associated protein 4 (CTLA-4), programmed death-1 (PD-1), T cell immunoglobulin and mucin domain 3 (TIM-3), lymphocyte-activation gene 3 (LAG-3), and T cell immunoreceptor with Ig and ITIM domains (TIGIT) are involved [96,97,98,99]. Tumor cells can evade immune cell-based eradication by engaging immunological checkpoint molecules with their ligands, leading to immune evasion of malignant cells [100]. In response to long-term antigen exposure, activated T cells overexpress immunosuppressive checkpoint molecules, rendering them exhausted [101]. When these immune checkpoint molecules interact with their ligands in malignancies, T cell-directed cytotoxicity is reduced [101]. As a result, ICIs act by activating effector T cell function and disrupt inhibitory signals [102]. The molecular mechanisms behind the lack of responsiveness of cancer patients to ICIs are analyzed in depth [103].

Initially, the limited response of cancer patients to ICIs can be attributed to the main characteristics of “immune-excluded” and “immune-desert” cancers [104,105]. Immune-infiltrated tumors contain abundant intratumoral T cells, immune-excluded tumors restrict immune cells to the tumor periphery, and immune desert tumors show minimal or no immune infiltration [104,105]. In particular, the lack of responsiveness of cancer patients to ICIs stems primarily from the low infiltration of tumor-associated antigen (TAA)-specific cytotoxic CD8^+^ T cells into the tumor microenvironment (TME) [106]. “Immune-excluded” cancers confine the cytotoxic T cells to the tumor’s periphery by preventing their entry into the tumor site due to obstacles within the TME [107,108]. For example, the increased infiltration of CD8^+^ T cells in the TME is an essential determinant of the improved clinical response to anti-PD-1 immunotherapies [109]. In addition, the TME also includes immunosuppressive cells such as tumor-associated macrophages (TAMs), T regulatory cells (Tregs), and myeloid-derived suppressor cells (MDSCs) and molecules that impair T-cell metabolism and cytotoxicity [110,111,112,113,114].

Second, resistance is reinforced by a low tumor mutational burden (ΤΜΒ) [115,116,117,118]. Interestingly, a high TMB has been associated with improved efficacy of PD-1 and PD-L1 inhibitors across cancers, including melanoma, lung cancer, and MSI-positive colorectal cancer [119,120].

Third, the resistance to ICI-based immunotherapy can be significantly influenced by antigenic escape through the insufficient presentation of tumor antigens. T-cell activation can be hindered by tumor cell-intrinsic changes, including modifications to immune-regulatory signaling pathways, downregulation of MHC-I, and low levels of expression of molecules involved in antigen-processing machinery [121]. In particular, the lack of responsiveness of cancer patients to ICIs may be related to abnormalities in interferon signaling pathways, impaired antigen cross-presentation, or decreased HLA expression, thereby resulting in ineffective presentation of tumor antigens by antigen-presenting cells (APCs) [121]. In this context, another example of resistance to ICI is the inactivation of JAK/STAT signaling, leading to downregulation of major histocompatibility complex-1 (MHC-I) and low PD-L1 expression, resulting in eventual resistance to PD-1 and CTLA-4 inhibition [122].

As a result, the tumor’s immunophenotypic state—based on T-cell infiltration, antigenicity, and mutation burden—is essential for predicting ICI response.

Another important biomarker for predicting response to ICI treatment is the frequency of PD-L1 expression on tumor cells [123]. It has been demonstrated that tumors with lower levels of PD-L1 expression typically exhibit worse clinical outcomes following ICI treatment when compared to those with greater ligand levels [124]. Tumor cells can also evade PD-1/PD-L1 or CTLA-4 inhibition by overexpressing other inhibitory checkpoints such as LAG-3, TIM-3, TIGIT, and V-domain Ig suppressor of T-cell activation (VISTA) [125]. Recent research illustrates how hypoxia, lactate formation, and nutritional depletion can interfere with T-cell energy metabolism, thereby limiting ICI effectiveness [126]. The epigenetic landscape of tumor cells can regulate antigen presentation patterns, establishing persistent immune evasion of tumor cells [127].

Last but not least, deficiencies in the DNA mismatch repair (MMR) system (dMMR) and microsatellite instability (MSI) have also been identified as essential determinants of ICI response. While microsatellite-stable (MSS) tumors maintain proficient MMR (pMMR) and show lower mutation loads, microsatellite-instable (MSI) colorectal cancers are caused by deficiencies in the DNA mismatch repair (MMR) system (dMMR), resulting in a high mutation rate [128]. In particular, ICI therapy can improve disease management in patients with dMMR/MSI colorectal cancer (CRC), emphasizing the predictive importance of MSI-associated neoantigenicity [129,130]. When combined, these biomarkers—PD-L1 expression, TIL prevalence, TMB, and dMMR/MSI status—will be crucial for enhancing patient classification and potentiating the therapeutic efficacy of ICI agents.

### 4.2. Combination of Immune Checkpoint Inhibitors (ICIs) with Telomerase Peptide Vaccines

Although a significant and increasing number of cancer patients have experienced clinical benefits from ICIs, they do not elicit a clinical immune response in a substantial number of individuals, implying that understanding the comprehensive mechanisms underlying resistance to ICIs is essential [95]. The role of telomerase peptide vaccines is to strengthen the pre-existing tumor immune response, increasing the infiltration and activation of cancer-specific T cells, especially in non-immunogenic tumors. This phenomenon can be potentiated with the use of ICIs to lessen the tumor’s immune suppressive mechanisms. The fundamental idea underlying the combination of telomerase vaccines with ICIs relies on inducing the proliferation of hTERT-specific T cells with telomerase peptide vaccines, while ICIs counteract T-cell-inhibitory signals, thereby mitigating T-cell exhaustion [78,131]. Indeed, telomerase peptide vaccines have a significant clinical impact when used with ICIs by (i) increasing the heterogeneity of hTERT-specific T cells, (ii) preserving the function of effector and cytotoxic T cells, and (iii) regulating the TME to allow T-cell infiltration [78,131].

A key aspect of current immunotherapy is the use of ICIs, which can reverse local intratumor immunosuppression by inhibiting the PD-1/PD-L1 signaling cascade [132]. Patients with immunogenic tumors, characterized by a substantial presence of tumor-specific T-cell infiltrates (TILs), exhibit favorable responses to ICIs. Conversely, patients with non-immunogenic tumors that are either completely or minimally infiltrated by TILs demonstrate no response to ICIs [133,134]. Additionally, the TMB reflects the load of neoantigens generated by gene mutations during oncogenesis and is positively correlated with increased tumor immunogenicity. In fact, tumors with a high TMB are the most immunogenic and responsive to ICIs [135].

While telomerase peptide vaccines alone may be insufficient in heavily immunosuppressed tumor environments, the combination of telomerase peptide vaccines with ICIs has emerged as a highly promising strategy that further enhances immunosurveillance. The combination of telomerase vaccines with ICIs greatly increases therapeutic efficacy through increasing intratumoral T-cell infiltration by restoring dysfunctional T cells, facilitating long-lasting responses. Telomerase peptide vaccines have been verified in preclinical studies in combination with ICIs, but the GX301 or Vx-001 telomerase vaccines have not. In addition to their combination with immunotherapy, telomerase peptide vaccines can be combined with either adoptive cell therapy (ACT), surgery, radiotherapy, or chemotherapy. Conventional methods of treatment, such as surgery, hormone treatment, radiation, and chemotherapy, have proved ineffective due to their high toxicity for patients, increasing mortality [136]. When chemotherapy is used, tumor antigens are released, which helps the immune system eradicate tumor cells. Specific immune responses against tumor-associated antigens, such as hTERT, can be enhanced by combining chemotherapy with telomerase-based vaccines, thereby increasing their effectiveness. When radiotherapy is applied, there is an increased release of telomerase antigen epitopes from the dying tumor cells, making telomerase peptide vaccines more likely to generate meaningful clinical outcomes. In this review, the existing knowledge regarding ongoing clinical trials is summarized. Future randomized studies are needed to identify which tumor types, treatment lines, and biomarker profiles benefit most from these combination strategies.

Undoubtedly, the generation of GX301, Vx-001, UV1, GV1001, and UCP-Vax demonstrates how telomerase peptide vaccines have revolutionized cancer therapy over the past 20 years. Despite the fact that hTERT is a key antigenic component, the clinical efficacy of telomerase peptide vaccines differs significantly because of small variations in antigen targets, HLA typing, the induction of CD4^+^ T and CD8^+^ T immune responses, the use of adjuvants, and synergy with ICIs. These differences in telomerase peptide vaccines are apparent in Table 2.

### 4.3. The Clinical Efficacy of the UV1 Telomerase Peptide Vaccine and Its Combinations

UV1 is a telomerase SLP-based vaccine consisting of three long peptides (15–30 amino acids), corresponding to the region of 54 amino acids in the catalytic unit of the human telomerase reverse transcriptase (hTERT) [137]. In clinical settings, the UV1 telomerase vaccine has emerged as a promising treatment option for several malignancies such as NSCLC [79]. Most NSCLC patients exhibited upregulated T-cell responses to the UV1 telomerase vaccine [79]. The UV1-treated patients showed increased recruitment of CD4^+^ T helper cells and increased release of Th1-associated cytokines [79]. Regarding the clinical benefit of the UV1 telomerase vaccine, it has been highlighted that long-term cancer survival was observed when the UV1 telomerase vaccine was administered, suggesting that UV1 can increase cancer patient survival [40]. Consistent with the above, cancer patients who exhibited an induced CD4^+^ T helper immune response presented a higher OS rate [138]. Another Phase I/IIa clinical study confirmed the immunogenicity of the UV1 telomerase vaccine [139]. In particular, this study included twenty-two patients with newly diagnosed metastatic hormone-naïve prostate cancer (mPC) who had begun androgen deprivation treatment (ADT) and had no visceral metastases [139]. At the end of the nine-month monitoring time frame, 17 patients had clinically stable disease, with specific immune responses induced in a large proportion of patients, regardless of HLA type [139]. Treatment of mPC patients with UV1 and GM-CSF resulted in few adverse events, which were predominantly grade 1, with the most frequent being injection-site pruritus [139]. In line with this, serious adverse events related to UV1 and/or GM-CSF in the aforementioned Phase I/IIa clinical trial included an anaphylactic reaction in two patients and thrombocytopenia in one patient [139].

### 4.4. Combination of UV1 Telomerase Peptide Vaccine with Immune Checkpoint Inhibitors (ICIs)

To assess the safety and effectiveness of UV1 in conjunction with single or multiple ICIs, various clinical trials have been conducted. In particular, ipilimumab or pembrolizumab in melanoma patients (NCT02275416 and NCT03538314, respectively) and ipilimumab combined with nivolumab (anti-PD1 agent) in patients with mesothelioma (NCT04300244) or melanoma (NCT04382664) are examples of ICIs whose clinical value has been evaluated in combination with the UV1 telomerase vaccine. In this review, the findings from ongoing clinical trials combining ICIs with the UV1 telomerase vaccine are discussed.

Initially, a Phase I clinical study combined the UV1 telomerase peptide vaccine with pembrolizumab (an anti-PD-1 agent) as a first-line therapy in patients with advanced melanoma [140]. In response to this combination therapeutic scheme, patients with treated melanoma showed a clinical benefit linked to the induction of CD4^+^ and CD8^+^ immune responses in an HLA-unrestricted manner [140]. The results of this phase I clinical trial also confirmed the safety of this combination therapeutic scheme in patients with advanced melanoma [140]. Fatigue, diarrhea, injection-site response, and pyrexia were the most common grade 1 or 2 adverse events [140]. Later, patients with metastatic melanoma were treated with the UV1 telomerase vaccine in combination with ipilimumab (a monoclonal antibody targeting CTLA-4), demonstrating the safety and clinical efficacy of the UV1 telomerase vaccine in this Phase I/IIa single-center trial (CT02275416) [141]. The mechanism of action of the UV1 telomerase vaccine was based on eliciting the activation of CD4^+^ T helper cells and the increased IFN-γ generation [141]. The clinical benefits of the combination therapeutic scheme demonstrated synergistic modes of action of the UV1 telomerase vaccine and ipilimumab [141]. Likewise, melanoma patients treated with the UV1 telomerase vaccine in combination with ipilimumab showed a significant improvement in clinical response [90]. Indeed, telomerase immunization triggered strong CD4^+^/CD8^+^ T-cell responses by promoting a heterogeneous T-cell receptor (TCR) repertoire, exposing an increasing number of telomerase epitopes bound by TCR clones and targeting multiple tumor subpopulations, thereby mediating more substantial and more effective tumor eradication [90]. As a result, this study provided convincing evidence that the UV1 telomerase vaccine in conjunction with ipilimumab sustained a persistent, long-term CD4^+^/CD8^+^ T-cell immune response against different tumor subpopulations in advanced melanoma patients by promoting the diversification of the TCR repertoire [90].

Consistent with this, another Phase I multicenter study (NCT03538314) showed that patients with advanced melanoma treated with the UV1 telomerase vaccine in combination with pembrolizumab (monoclonal antibody targeting PD-1) did not experience severe side effects, suggesting the safety and tolerability of this combinatorial therapeutic scheme [142]. Following this, the combination of pembrolizumab with the UV1 telomerase vaccine proved effective for treating advanced melanoma, an example of a less immunogenic tumor, in a Phase I clinical trial [143]. Evaluating the safety of this combination therapeutic scheme was the main purpose of this study, which also measured progression-free survival (PFS), overall survival (OS), and the objective response rate (ORR) in thirty advanced melanoma patients following the combination therapeutic scheme [143]. In particular, the melanoma patients showed increased survival rates associated with induced immune responses [143]. In particular, the results showed that the OS rates for treated cancer patients were 86.7% and 73.3% at 1 and 2 years, respectively [143]. Notably, the ORR among treated cancer patients was 56.7%, with 33.3% of responses complete [143]. However, a small percentage of melanoma patients (20%) experienced only grade 3 adverse events, which included moderate injection-site responses [143]. A recent Phase II study found that patients with advanced melanoma treated with the UV1 telomerase peptide vaccine plus ipilimumab (anti-CTLA-4 agent) and nivolumab (anti-PD-1 agent) did not experience any clinical benefit [144]. Accordingly, recent clinical data from UV1 plus pembrolizumab in certain solid tumors revealed that this combination scheme did not substantially enhance survival benefit compared to pembrolizumab alone, suggesting that the clinical advantage could rely on tumor type, disease state, and immune system setting [145].

Last but not least, the combination of the UV1 telomerase vaccine with monoclonal antibodies targeting CTLA-4 and PD-1 has been shown to elicit a strong immune response in malignant pleural mesothelioma [146]. In particular, the combination of the UV1 telomerase vaccine with ipilimumab and nivolumab increased the intensity of the immune response without improving survival rates in patients with malignant pleural mesothelioma [146]. The aforementioned results provide convincing evidence for the combinatorial use of the UV1 telomerase vaccine and ICIs to enhance cancer treatment in patients.

In summary, UV1 is one of the most immunogenically effective telomerase vaccines developed. However, the UV1-induced immune response by itself is insufficient to ensure clinical benefit in treated cancer patients since the therapeutic effect of the UV1 telomerase vaccine is affected by tumor type, tumor stage, immunological context, and combination therapy.

### 4.5. The Clinical Efficacy of GX301 Telomerase Peptide Vaccine

Another telomerase SLP-based vaccine is the GX301 telomerase vaccine. The multipeptide GX301 vaccine is composed of four hTERT-derived peptides of 540–548 amino acids, 611–626 amino acids, 672–686 amino acids, and 766–780 amino acids [83]. In a preclinical setting, GX301’s immunogenicity was proved in vitro through the induction of T helper and T cytotoxic cells [83]. In clinical settings, the GX301 multipeptide vaccine has been shown to harbor significant advantages over single-peptide vaccines in terms of immunogenicity. First, the GX301 vaccine is compatible with MHC class I and II molecules, thereby boosting the activation of helper CD4^+^ and cytotoxic CD8^+^ T cells [83]. For this reason, the multipeptide Gx301 telomerase vaccine elicits a stronger immune response and more responders than a single peptide, suggesting that GX301 is a good example of a multi-peptide vaccine strategy [83]. When each peptide was administered independently, all patients showed immune responses to at least one peptide, confirming the peptide-specific responses and patients’ specificity for each peptide [83]. In addition, immunological tolerance does not appear to significantly affect immune responses elicited by the GX301 vaccine, nor does it increase the risk for autoimmunity, given that telomerase is an endogenous antigen [83]. In addition, it has been reported that the telomerase vaccine GX301 is administered with adjuvants such as Montanide ISA-51 and an immune-inducing compound such as imiquimod [83]. Montanide has been reported to form a water-in-oil emulsion that facilitates intradermal DC peptide uptake and protects peptides from tissue proteases. It also stimulates innate immune cells to release IFNγ [147]. Similarly, imiquimod, a potent activator of Toll-like receptors (7 and 8), promotes DC activation and maturation [147]. As a result, these two adjuvants appear to collaborate to enhance the uptake and presentation of peptide vaccines via complementary mechanisms. In particular, 500 μg of each peptide (dissolved in Montanide ISA-51) of the GX301 telomerase vaccine was injected intradermally into the abdominal skin of patients with renal or prostate cancer in phase I/II clinical trials [83]. To assess the immune-inducing effects of the GX301 telomerase vaccine, ELISPOT and intracellular cytokine staining by flow cytometry were used to evaluate peptide-specific immune responses. The findings demonstrated that each peptide elicited distinct immune responses in specific individuals, with distinct responder panels across the peptides. In particular, the number of responders and the immune responses to multiplex peptides were greater than the responses to single peptides, suggesting that multi-peptide vaccines are more effective than single-peptide vaccines [83]. As a result, strong immunogenicity was elicited by GX301 administration because most treated patients had antigen-specific CD4^+^ and/or CD8^+^ T-cell responses against at least one hTERT-derived peptide [83,148]. Despite these robust immune responses, clinical efficacy was limited. In a recent randomized Phase II open-label clinical trial showed the immunogenicity and safety of the GX301 vaccine, which was administered to ninety-eight patients with metastatic castration-resistant prostate cancer (mCRPC) in three different schemes [82], 95% of treated cancer patients developed at least one specific immune response against cancer cells that persisted for an extended period [82]. However, no clinical benefit was observed in GX301-treated cancer patients, since the overall survival of GX301-treated cancer patients did not present any difference from that of patients receiving standard-of-care therapy alone [82]. Regarding the safety profile, GX301 was observed to be well-tolerated with no significant adverse effects [82]. Although the GX301 vaccine induced immune responses in a safe manner, GX301-induced immunogenicity did not result in a survival advantage among cancer patients.

### 4.6. The Clinical Use of the Vx-001 Telomerase Peptide Vaccine

Another SLP telomerase-based vaccine is the Vx-001 telomerase vaccine [149,150]. The functional part of the cryptic peptide involved in this telomerase vaccine is concealed within the protein [149,150]. Thus, the first vaccine based on the “optimized cryptic telomerase epitope” is Vx-001. In particular, the Vx-001 vaccine includes two specific nine-amino-acid peptides, comprising the cryptic TERT peptide and its optimized version, in which tyrosine is substituted for the first amino acid, increasing its binding affinity for human leukocyte antigen (HLA)-I, rendering this Vx-001 telomerase vaccine able to induce a strong immune response to cancer cells [135,151,152].

To better understand the cryptic peptide involved in the telomerase vaccine, it is worth noting that peptides produced via antigen processing can be roughly divided into two groups, dominant and cryptic peptides, based on their affinity for MHC molecules. This classification affects each peptide’s potential for successful cross-presentation by its APCs. During thymic selection, T cells with a high affinity for self-peptide/self-MHC complexes are clonally eliminated. Since cryptic peptides bypass thymic self-tolerance, the immune system recognizes them as non-self. However, cryptic peptides are ineffective at eliciting an anti-cancer immune response in their original form due to their low MHC affinity, rendering them very weakly immunogenic [151]. Due to poor MHC loading or restricted surface presentation, these subdominant or low-affinity cryptic hTERT epitopes lead to inadequate T-cell activation. Such cryptic epitopes may induce partial immunological tolerance rather than strong T-cell cytotoxicity, thereby reducing the clinical efficacy of the Vx-001 telomerase vaccine. Because of cryptic peptides’ low level of expression, cryptic peptides derived from self-antigens are regarded by the immune system as non-self and not associated with immunological tolerance or risk of autoimmunity [151]. As a result, these weakly immunogenic cryptic peptides cannot be used in their original form to elicit an effective anti-cancer immune response. By attaching selectively chosen amino acids that bind to HLA-I to the primary and secondary attachment sites of the cryptic peptide sequence, the immunogenicity of these peptides can be increased. In this regard, amino acid modifications generate “optimized cryptic peptides” that are highly immunogenic [151].

In a clinical setting, the Vx-001 telomerase vaccine elicited a TERT-specific T-cell immune response, which was associated with longer survival, making it a promising option for cancer immunotherapy [135]. In that study, the Vx001 telomerase vaccine was effective in NSCLC patients who received the aforementioned treatment modality. In particular, they mounted immune responses in an HLA-A*0201-specific manner in NSCLC patients, prolonging their survival [135]. Subsequently, the T-cell immune response to Vx-001 was evaluated in patients with advanced solid tumors that were resistant to chemotherapy who received subcutaneous Vx-001 injections [135]. Intracellular cytokine staining tests and IFN-γ and perforin ELISpot assays were used to evaluate the specific immune response in these patients [135]. The study results highlighted the emergence of TERT-reactive T cells in patients following Vx-001 telomerase vaccination [135]. Interestingly, immune responses were observed regardless of disease stage or pre-vaccination status [135]. Patients who developed a strong immune response showed a significant survival advantage compared to post-vaccination non-responders [135]. Although the Vx-001 telomerase vaccine did not extend the lifespan of patients with NSCLC, it elicited an immune response, as evidenced by the activation of cytotoxic CD8+ T cells [135]. Mavroudis et al. highlighted the expansion of T cells following prior administration of the optimized hTERT peptide (p572Y) in patients with progressive and chemotherapy-refractory tumors [153]. In a Phase I clinical trial, patients with advanced NSCLC were treated with the optimized telomerase epitope (TERT572Y) [154]. Τhe researchers used the optimized TERT572Y peptide, in which the substitution of leucine (L) with tyrosine (Y) increases binding to HLA-A*0201 molecules. The optimization of a cryptic telomerase peptide improved HLA-binding stability and T-cell activation, converting a weakly immunogenic self-peptide into a potent vaccine epitope [154]. In this manner, patients treated with Vx-001, an optimized telomerase epitope (TERT572Y), developed a robust CD8^+^ T-cell immune response associated with prolonged survival [154]. In addition, patients treated with Vx-001 were evaluated for their immune and clinical responses in advanced solid tumors [155]. In particular, the TERT-specific immune responses induced by the well-tolerated Vx-001 vaccine were strongly associated with improved clinical outcomes [155]. Regarding the time window of Vx-001 immunogenicity, Vx-001 induced hTERT-reactive T cells in a significant percentage of cancer patients after six doses of telomerase vaccine and in half of the cancer patients following two doses of telomerase vaccine [155]. In another clinical study, Vx-001 was evaluated in patients with various advanced solid tumors in a Phase II clinical trial. Following the second and sixth vaccinations, 55% and 70% of patients demonstrated hTERT-specific T cell immune responses, which were associated with improved clinical outcomes (longer PFS and OS rates) compared with non-responders [150]. In a Phase 1/2 two-arm study, ex vivo anti-CD3/anti-CD28 costimulated autologous T cells were administered to myeloma patients two days after autologous stem cell transplantation [156]. The treatment of melanoma patients focused on immunization with the Vx-001 telomerase multipeptide vaccine and the antiapoptotic protein survivin [156]. For immunizing myeloma patients with the Vx-001 multipeptide vaccine, two optimized cryptic peptides (p572Y and p988Y) and the hTERT p540 peptide were used [156]. According to this study, some patients who developed a sufficient immune response after vaccination still did not benefit clinically from Vx-001 [156].

Even though there are currently no reported findings in the literature, two additional multi-epitope vaccines constructed from optimized cryptic peptides were recently introduced: Vbx-016, which focuses on four optimized cryptic peptides derived from four tumor antigens (CEA, MAGE, TERT, and HER-2/neu), and Vx-006, consisting of three designed cryptic peptides obtained from three different TAA (MAGE, TERT, and HER-2/neu) [157].

### 4.7. The Clinical Use of the GV1001 Telomerase Vaccine

The GV1001 MHC class II-restricted peptide vaccine has been designed to cover 16 amino acids that are included in the human telomerase reverse transcriptase (hTERT) catalytic subunit (mapping the 611–626 amino acid sequence of hTERT) [158]. GV1001 was initially developed as a prevalent telomerase peptide vaccine for the treatment of cancers [159]. In a preclinical setting, the GV1001 telomerase vaccine attenuated tumor growth by downregulating hypoxia-related factors and angiogenic factors. The in vitro effectiveness of the GV1001 vaccine was observed in human T-cell leukemia, human cervical adenocarcinoma, and human breast adenocarcinoma [160].

In a clinical setting, the GV1001 telomerase vaccine has proven safe in multiple clinical trials for hepatocellular carcinoma (HCC) [161], non-small-cell lung cancer (NSCLC) [84], pancreatic cancer [162], prostate cancer [163,164] and melanoma [165]. The GV1001 telomerase has been shown to exert its anti-tumor effect once the adjuvants and endogenous processing of the telomerase peptide are accomplished. For this reason, the GV1001 vaccine is used alongside adjuvants such as GM-CSF or TLR-7 [166]. For example, GM-CSF has been extensively used in cancer immunotherapy as an immune-boosting adjuvant, as it promotes DC infiltration and maturation and activates neutrophils, macrophages, and NK cells. According to another phase I clinical trial, the combination of GV1001 with another adjuvant like imiquimod was well-tolerated in patients with non-resectable pancreatic cancer. Those findings confirmed that imiquimod can be used as an adjuvant for peptide vaccination alone or in combination with GM-CSF [162]. The aforementioned study was conducted to elucidate the reason for imiquimod’s superior inflammatory response compared to GM-CSF, which is attributed to the activation of TLR-7 on APCs [162].

The GV1001 telomerase vaccine has emerged as a universal cancer vaccine because it can be administered without requiring HLA typing and induces strong immunogenicity. For example, twenty-six patients with non-small-cell lung cancer (NSCLC) enrolled in Phase I/II trials demonstrated strong T-cell immune responses following treatment with the GV1001 telomerase vaccine [167]. After immunization with the single hTERT peptide GV1001 vaccine, a comprehensive immunological analysis of T-cell responses against telomerase (hTERT) was performed, comparing clinically responsive individuals with non-responders [88]. Interestingly, a heterogeneous immune response to this GV1001 single-peptide telomerase vaccine was observed, as CD4^+^ T-cell clones specific for the telomerase antigen epitope were detected in lung cancer patients who were in complete remission after immunization, highlighting the positive relationship between the GV1001 telomerase’s clinical benefit and the activated immune response [88]. In addition, the GV1001 telomerase vaccine induced an “epitope spreading effect” in the immune response, which was specific not only to a particular peptide epitope but also to structurally unrelated epitopes inside the hTERT molecule [88]. Clinically, patients with lung cancer developed T-cell responses against many unrelated hTERT epitopes that were not part of the vaccine peptide, representing typical intramolecular epitope spreading [88]. Interestingly, this intramolecular spreading phenomenon was observed in clinical responders but not in patients who had only an immunological response to the vaccine peptide without clinical benefit [88]. The molecular mechanism underlying the beneficial effect of the GV1001 telomerase vaccine relies on triggering CD4^+^ T helper and CD8^+^ T cytotoxic cell responses in the immune system’s fight against cancer cells, resulting in long-term T-cell memory [168]. As a result, the aforementioned research supports that the GV1001-mediated immune response is linked to disease control in immune responders.

In another Phase I/II study, patients with inoperable pancreatic cancer with poor prognosis were enrolled and treated with the GV1001 telomerase vaccine, demonstrating that it is safe and immunogenic, with a significant overall survival benefit [162]. In particular, the lifespan of patients with incurable pancreatic cancer was increased and was associated with the activation of an immune response [162]. In another recent study, GV1001-induced immune responses were the best predictive indicator of clinical benefit in advanced pancreatic and pulmonary cancers. In particular, it was highlighted that patients who developed strong GV1001-specific T-cell responses exhibited high IFN-γ/IL-10 ratios and polyfunctional cytokine patterns, experienced longer survival, and sustained clinical benefits [168,169]. Interestingly, not all immune responders benefit clinically, implying that the intensity and resilience of the immune response are crucial for predicting clinical outcomes [168].

In addition to the above, GV1001 has been reported to penetrate tumor cells and downregulate vascular endothelial growth factor (VEGF), hypoxia-inducible factor (HIF)-1, heat shock protein (HSP) 90, and HSP70, thereby enhancing its anti-tumor effect [160,170]. In particular, GV1001 has been demonstrated to decrease the quantity of HSPs both within and on the cell surface by crossing the cell membrane and localizing in the cytoplasm. GV1001 has been shown to reduce the amount of HSPs both inside and on the cell surface by penetrating the cell membrane and localizing in the cytoplasm. It also decreases the expression of VEGF, HSP90, HSP70, and HIF-1α in hypoxic tumors. Since GV1001 is a cell-penetrating peptide, it is proposed to have strong anticancer effects [160,170]. In this manner, GV1001 appears to be especially effective in tumors with high angiogenic activity, taking into consideration that GV1001 has anti-angiogenic effects by inhibiting the VEGF-A/VEGFR-2 pathways and matrix metalloproteinase expression, leading to reduced tumor growth and invasion in preclinical models. This indicates that GV1001-based treatments could be more beneficial for patients with malignancies driven by angiogenesis [160,170]. However, patients with cutaneous T cell lymphoma (CTCL) did not respond objectively to the GV1001 telomerase vaccine, implying its ineffectiveness [171]. Interestingly, 16% of patients treated with the GV001 telomerase vaccine did not show the expected increase in leukocyte infiltration at tumor sites [171].

Last, GV1001 has shown antioxidant and neuroprotective properties. These effects are realized by neutralizing reactive oxygen species (ROS) and reducing death signals [172]. In particular, GV1001 induced apoptosis in cancer cells in renal cell carcinoma by inhibiting angiogenesis [173]. Additionally, GV1001 was shown to exhibit antiviral activity [174,175], anti-inflammatory effects [176,177,178], and protective effects against β-amyloid-induced neurotoxicity in the central nervous system [179]. In summary, GV1001 shows a good safety profile and modest immunogenicity in pancreatic and lung cancer, highlighting the challenge of peptide monotherapy in immunologically cold malignancies.

### 4.8. Combination of GV1001 Telomerase Vaccine with Immune Checkpoint Inhibitors (ICIs)

Several studies have examined the clinical and immune efficacy of ICIs combined with the GV1001 telomerase vaccine across different tumor types. Results from clinical studies examining the GV1001 telomerase vaccine combined with immunotherapy, radiation, or chemotherapy vary depending on the specific tumor type and treatment armamentarium. Initially, forty-eight patients with advanced pancreatic cancer were treated with GV1001 telomerase vaccine and GM-CSF in a Phase I/II study using a three-dose scheme [162]. Sixty-three percent of the evaluated patients exhibited immune responses, with the highest rate (75%) in the intermediate-dose group [162]. Interestingly, the median survival of patients treated with an intermediate dose of the GV1001 telomerase vaccine was significantly longer than that of the other groups, suggesting a link between extended survival and immune activation [162].

According to preclinical evidence on pancreatic ductal adenocarcinoma, GV1001 in combination with gemcitabine eliminates tumor cells by activating death pathways and significantly reducing tumor tissue scarring [180]. In the clinical setting, GV1001 was initially evaluated in patients with locally advanced or metastatic pancreatic cancer to compare chemotherapy with or without GV1001 in a Phase III trial [181]. In patients with locally advanced or metastatic pancreatic cancer, the safety and effectiveness of sequential or concurrent administration of the telomerase vaccine (GV1001) in combination with chemotherapy (gemcitabine and capecitabine) were evaluated [181]. The results of the TeloVac study (Phase 3 clinical trial) highlighted that the introduction of the GV1001 vaccine did not improve the clinical/survival status of cancer patients, as the median OS of the chemotherapy group did not significantly differ from that of the concurrent chemoimmunotherapy group or the sequential chemoimmunotherapy group [181]. Interestingly, immune-mediated responses were observed, but they did not lead to clinical improvement, with no survival benefit for participants whose immune systems responded [181]. In addition, patients in all groups presented similar grade 3–4 side effects, including pain, fatigue, and neutropenia [181], suggesting that the combination of the telomerase vaccine (GV1001) with chemotherapy was well-tolerated and safe, even without survival benefit [181]. In another study, the inflammatory response was proven to be attenuated in pancreatic ductal adenocarcinoma patients following treatment with gemcitabine and the GV1001 telomerase vaccine [180]. In particular, tumor necrosis factor (TNF)-α, IL-6, and IL-1β levels were reduced in the gemcitabine and GV1001 group, whereas their levels were increased in the gemcitabine alone group. When GV1001 and gemcitabine were administered together, there was a significant reduction in tumor tissue fibrosis and tumor cell death [180]. In addition, the GV1001 telomerase peptide vaccine was evaluated in conjunction with granulocyte–monocyte colony-stimulating factor (GM-CSF) and gemcitabine as first-line therapy in a research study examining safety and immunogenicity in patients with incurable pancreatic cancer [159]. Assays, including delayed-type hypersensitivity (DTH), enzyme-linked immunospot (ELISPOT), and cytokine secretion assays, showed an induced GV1001-specific immune response. Still, it was not sufficient to support the efficacy of the aforementioned therapeutic scheme. Chemotherapy with the telomerase vaccine (GV1001) was safe, although the immune responses were modest and transient [159].

A recent Phase III trial in patients with high eotaxin levels revealed that GV1001 plus chemotherapy improved median overall survival (OS) (11.3 vs. 7.5 months) and progression-free survival (PFS) rates (7.3 vs. 4.5 months) [182]. Since eotaxin can boost T cell infiltration into the tumor by acting as a chemoattractant, patients who received GV1001 in combination with gemcitabine/capecitabine had higher survival when their serum eotaxin levels were high. These findings led to the approval of a GV1001 telomerase vaccine for patients with elevated serum eotaxin levels and locally advanced or metastatic pancreatic cancer [182]. In patients with advanced PDAC and eotaxin-rich tumors, GV1001 plus gemcitabine/capecitabine improved overall survival (OS) and time to progression (TTP) compared with gemcitabine/capecitabine alone [182]. In a recent study, the GV1001 telomerase vaccine was beneficial when combined with gemcitabine/capecitabine. In patients with advanced pancreatic ductal adenocarcinoma (PDAC) and high eotaxin levels, the GV1001 telomerase vaccine, in combination with gemcitabine/capecitabine, appeared effective, improving OS and TTP compared to gemcitabine/capecitabine alone [182]. Nevertheless, not all patients with elevated serum eotaxin levels responded positively to the GV1001 telomerase peptide vaccine [182]. The highly immunosuppressive TME of pancreatic cancer appears to be the primary determinant of the reduced clinical potential of the GV1001 telomerase-based vaccine, leading to its ineffectiveness and the progression of pancreatic tumors [183]. Meanwhile, GV1001 vaccination triggered GV1001-specific immune responses when combined with chemoradiotherapy in NSCLC patients [84,184]. Compared to nonresponders, immune responders had longer median progression-free survival (PFS) (371 vs. 182 days) and overall survival (OS) (19 vs. 3.5 months). Durable T-cell memory responses appeared to be maintained among long-term survivors [84].

In greater detail, a clinical Phase I/II trial demonstrated that the telomerase peptide vaccines GV1001 (which covers the 611–626 amino acid sequence of hTERT) and HR2822 (which covers the 540–548 amino acid sequence of hTERT) effectively elicited strong immune responses, considering that HR2822 is an HLA-A2-dependent telomerase epitope that triggers cytotoxic T lymphocytes [167]. In this trial, NSCLC patients who received the aforementioned combination of telomerase peptides experienced an improvement in their clinical response without any toxic side-effects [167]. In the clinical trial, one patient achieved a complete response (CR), and 86% of patients had an immunological response [167]. This clinical trial suggests that combination therapy with an hTERT peptide vaccine may be more effective than vaccination alone [167]. Interestingly, the GV1001 vaccine was administered after a week of docetaxel and radiation therapy for patients with incurable stage III NSCLC (CTN-2006) [84]. An 8-year follow-up on a previously reported Phase I/II trial (CTN-2000) was accomplished using two telomerase peptides (GV1001 and I540) [84]. The CTN-2006 trial, which tested the same vaccine, GV1001, as a maintenance therapy after chemoradiotherapy, showed a less Th1-polarized phenotype than those from the CTN-2000 trial, where patients received hTERT vaccination as monotherapy, thereby suggesting that the phenotype of vaccine-induced T cells and their anti-tumor activity may be influenced by disease stage and prior treatments [84]. Specifically, the treatment scheme was as follows: GV1001 vaccine after chemoradiotherapy (radiation plus weekly docetaxel). The trial evaluated the efficacy of two treatment regimens: one with radiation therapy and chemotherapy followed by GV1001 administration and another combining GV1001 with p540 peptides. The goal was to evaluate immune response, toxicity, and clinical benefit after GV1001 vaccination. The results showed that the GV1001 vaccine was well-tolerated and stimulated immune responses and strong memory in most patients with non-small-cell lung cancer (NSCLC) [84].

After four years, two telomerase peptide-based vaccines (GV1001 and I540) were proved to be effective in NSCLC patients [84]. In particular, a Phase II trial (CTN-2006) and an eight-year update on a previously published Phase I/II trial (CTN-2000) were conducted in NSCLC patients who were immunized with telomerase peptide-based vaccines and underwent chemoradiotherapy [84]. The GV1001 vaccine was administered after weekly applications of docetaxel and radiation therapy to stage III patients with NSCLC [84]. The results highlighted that telomerase peptide-based vaccine responders lived longer, with stronger elicited immune responses in a statistically significant manner compared to nonresponders [84]. Following GV1001 vaccination, the patients had high interferon-gamma (IFNγ) expression levels, as well as low expression levels of anti-inflammatory molecules, including IL-10, and exhibited lasting and persistent T-cell memory responses [84]. Although chemoradiotherapy combinations have been investigated in NSCLC, no clinical studies combining GV1001 with radiation alone have been observed [84].

Patients with cutaneous melanoma were treated with combinations of telomerase-derived peptides, GV1001 (hTERT: 611–626) and p540 (hTERT: 540–548), utilizing tuberculin PPD23 or GM-CSF as adjuvants [185]. In particular, delayed-type hypersensitivity (DTH) reactions, in vitro T cell proliferation assays, and cytotoxicity (51-Chromium release) assays were used to quantify peptide-specific immune responses. This combination of telomerase vaccine demonstrated anti-cancer activity by inducing T cell proliferation to lyse melanoma cells in patients with advanced melanoma. Interestingly, it was proposed that the immune response against mycobacterial peptides prevailed, thereby hindering the response against the hTERT peptide [185]. As a result, the combination of GV1001 telomerase with pembrolizumab is under investigation, with early-phase data suggesting safety and immunogenicity in melanoma. The GV1001 telomerase peptide-based vaccine was also examined in combination with the alkylating agent temozolomide in patients with melanoma [186]. GV1001 and temozolomide-mediated immune responses were observed in the vast majority of patients (78%). The results showed that survival rates at 1 and 2 years were higher than historical controls, being at 44% and 16%, respectively, suggesting that increased GV1001-induced immune responses were associated with longer survival in melanoma patients [186]. In patients with advanced hepatocellular carcinoma (HCC), the effect of chemotherapy (low-dose cyclophosphamide) in conjunction with the GV1001 telomerase vaccine was examined in a Phase II clinical trial [161]. Although almost half of the patients with advanced HCC showed stable illness six months after starting treatment, they did not experience a survival benefit with the activation of the immune response. In particular, no GV1001-specific immune responses were observed in HCC patients after GV1001 telomerase vaccine administration, and none of the HCC patients experienced full or partial improvement with this treatment option [161]. In patients with metastatic colorectal cancer who had not responded to first-line chemotherapy, researchers assessed the safety and effectiveness of GV1001 in conjunction with chemotherapy [187]. Metastatic colorectal cancer patients who received the GV1001 telomerase vaccine plus chemotherapy as a second-line therapy had a median OS of 12.8 months and a median PFS of 7.1 months, showing an illness control rate of 90.9% [187]. Nevertheless, there were few GV1001-specific immunological responses, with only 28% exhibiting antigen-specific T-cell proliferation and no positive delayed-type hypersensitivity reactions [187]. As a result, the addition of GV1001 immunization to chemotherapy was tolerated and linked with modest effectiveness outcomes, despite the lack of a discernible GV1001-specific immune response [187]. In conclusion, GV1001 is usually well-tolerated when combined with chemotherapy, immunotherapy, or chemoradiotherapy, thereby triggering clinical benefits and immunological responses, depending on tumor type and treatment scheme.

### 4.9. The Clinical Efficacy of the UCP-Vax Telomerase Vaccine and Its Combinations

UCP-Vax is a multi-peptide rather than a single telomerase vaccine. The antigen targets of the UCP-Vax telomerase vaccine are hTERT and survivin, along with additional tumor-specific antigens that are delivered via DPX lipid-based depots. From this perspective, the delivery of the UCP-Vax telomerase vaccine is superior to the methods used for the GX301, Vx-001, and GV1001 telomerase peptide vaccines.

In a clinical setting, a recent Phase Ib/IIa showed the therapeutic benefit, immunogenicity, and safety of the UCP-Vax telomerase vaccine in patients with refractory advanced non-small-cell lung cancer (NSCLC) [188]. In particular, UCP-Vax elicited strong and persistent CD4^+^ Τ immune responses, demonstrating that the widespread hTERT-derived peptides effectively activate T cells, even in individuals with weakened immunity. Interestingly, patients who exhibited vaccine-specific T-cell responses sustained their disease, implying a positive relationship between UCP-Vax’s clinical benefits and induced immunogenicity [188]. Regarding its safety profile, the UCP-Vax telomerase vaccine has been well-tolerated. However, the administration of UCP-Vax telomerase vaccine seemed to result in some common complications like fatigue, mild flu-like symptoms, and grade 1–2 injection-site responses [188]. All of these results provide evidence that the UCP-Vax telomerase vaccine induces significant immunological activation and a modest but substantial therapeutic effect with a favorable safety profile [188]. Consistent with this, a recent study highlighted that the UCP-Vax telomerase vaccine attenuated advanced NSCLC progression in patients by stimulating polyfunctional CD4^+^ T cell responses, generating a variety of cytokines, including TNF-α and IL-2, and inducing epitope spreading across a wide range of MHC class II molecules [87]. The therapeutic benefit of the UCP-Vax telomerase vaccine was also attributable to its capacity to elicit a greater antibody response to hTERT, suggesting that the vaccine-induced CD4^+^ T cell response and this vaccine-specific IgG response were synergistic [87].

Recently, researchers presented an application of high-performance liquid chromatography (HPLC) to identify the primary molecule and any breakdown products that develop over time [189]. The results of this study supported, for the first time, that isoAspUCP4 telomerase peptide was separated from the active telomerase peptide components and Imps on the chromatogram, emphasizing the physicochemical stability of the UCPvax telomerase vaccine, which was stored for two months without degradation [189].

Regarding combination schemes, several clinical studies have provided evidence of proof of concept that UCP-Vax’s effectiveness can be enhanced when combined with ICIs. Initially, NSCLC patients showed amplification of hTERT-specific CD4^+^ T helper cells following treatment with the UCP-Vax telomerase vaccine and chemotherapy [190]. In another cancer type, the VolATIL study assessed whether PD-L1 inhibition can enhance the clinical efficacy of the UCP-Vax telomerase vaccine in immune-evaded HPV-positive malignancies [86]. The safety and effectiveness of the UCP-Vax telomerase vaccine in combination with atezolizumab (an antibody targeting PD-L1) were demonstrated in HPV-positive cancers [86]. When the UCP-Vax and atezolizumab were used, T-cell infiltration increased; the percentages of exhausted T-cells were reduced, and enhanced presentation of tumor antigens was observed, which contributed to increased patient responses [86]. The combination of atezolizumab with UCP-Vax was well tolerated, with no adverse autoimmune events reported [86]. According to this study, combining the UCP-Vax telomerase vaccine with atezolizumab was proven safe and immunologically synergistic in HPV-positive malignancies through the induction of CD4^+^ T immune responses [86]. These findings encourage further research on telomerase-based vaccines with the capacity to induce CD4^+^ T immune responses as amplifiers of ICIs, particularly in malignancies with high levels of telomerase expression and viral oncogenesis.

A multicenter randomized Phase II trial (NCT05528952,TERTIO—PRODIGE 82) confirmed the immunological efficacy and clinical benefit of a combination therapy that included the UCP-Vax telomerase vaccine with atezolizumab and bevacizumab (VEGF inhibitor) for incurable hepatocellular carcinoma (HCC) [191]. The molecular mechanism underlying the action of ICI therapy combined with the UCP-Vax telomerase vaccine relied on activating T cells and preventing immunosuppression in the TME in unresectable HCC [191]. This combination therapy activated and expanded tumor-specific T cells, enhancing tumor immunity and bypassing the effects of immunosuppressive cells in patients with locally advanced, metastatic or unresectable HCC [191].

A Phase II trial evaluated UCP-Vax with or without temozolomide after chemoradiation for newly diagnosed glioblastoma [89]. Specifically, patients were injected with UCP-Vax subcutaneously, and it did not pass their blood–brain barrier (BBB). When the UCP-Vax telomerase vaccine plus temozolomide was given, TERT-specific CD4^+^ T cell responses were induced [89] after the cross-presentation of hTERT peptides in lymphoid tissues by antigen-presenting cells (APCs) [87]. Given that the BBB is often disrupted [192], the immunization of cancer patients with the UCP-Vax telomerase vaccine enhanced TERT-specific immune responses, thereby contributing to increased survival [89]. The main principle behind administering the UCP-Vax vaccine in glioblastoma is increased immune infiltration and not direct vaccine diffusion [89]. Regarding the adverse effects of the UCP-Vax telomerase vaccine, it was proven to be well-tolerated in patients with newly diagnosed glioblastoma and did not cause any severe adverse reactions [89].

In summary, UCP-Vax provides a clinical impact by triggering strong immune responses in NSCLC, glioblastoma, and HPV-positive cancers when combined with immunotherapy or chemotherapy. In this way, immune responders often achieve higher rates of disease control and longer survival following treatment with the UCP-Vax telomerase vaccine.

A comprehensive summary of the clinical data on telomerase-based vaccines for various cancer types is provided in Table 3. It summarizes important clinical outcomes, vaccine-induced immunogenicity, and safety profiles in cancer patients treated with different telomerase vaccines, such as UV1, GV1001, GX301, Vx-001, and UCP-Vax, and combination strategies with immune checkpoint inhibitors or conventional therapies. In particular, the table highlights that telomerase vaccines often display good safety and immunogenicity, inducing hTERT-specific CD4^+^ and/or CD8^+^ T-cell responses associated with clinical benefit in a variety of cancers, such as NSCLC, melanoma, glioblastoma, pancreatic cancer, hepatocellular carcinoma, and cancers linked to HPV (Table 3). Importantly, the clinical benefit of telomerase vaccines becomes evident in immune responders and in cancer patients treated with combination schemes, emphasizing the need to combine telomerase vaccines with conventional chemotherapy or ICIs to maximize clinical efficacy.

## 5. The Dendritic Cell (DC)-Based Telomerase Vaccine

Cell-based technology is among the oldest methods for developing anti-cancer vaccines. These vaccines use immune cells or whole cells, which are often derived from a patient’s tumor [193,194]. In cell-based hTERT-targeted vaccines, the cells can be dendritic cells (DCs), tumor-infiltrated leukocytes (TILs), T-cell receptor-engineered T cells (TCR-T), chimeric antigen receptor T cells (CAR-T), or bispecific T-cell engagers (BiTEs) [195].

DCs have been a cornerstone of cancer immunotherapy in recent years, underscoring that DC-based vaccines are at the forefront of this field. In a molecular setting, increased TAA secretion is driven by increased cancer cell death. The released TAAs are engulfed by DCs, which degrade them into long peptides. T helper cell activation can occur when DCs present TAAs to the T cell receptor (TCR) of T cells via MHC class II molecules. In addition, DC vaccines are competent at presenting antigens via the MHC class I molecules, thereby triggering strong cytotoxic T lymphocyte responses [196].

The development of DC vaccines has progressed through three generations. The first generation, which had limited clinical success, primed DCs with messenger RNA or tumor-associated antigens (TAAs) administered ex vivo. Monocyte-derived DCs (MoDCs), peripheral blood mononuclear cells (PBMCs), or CD34^+^ hematopoietic progenitors were used to create the first generation of DC vaccines ex vivo [197,198]. In the first generation of DC-based hTERT-targeting vaccines, there were two primary methods for loading hTERT on DC vaccines: first, the in vitro incubation of DCs with the hTERT antigen epitope and a subsequent increase in antigen-presenting capacity [199], and second, hTERT mRNA overexpression in DCs [8,200]. To counteract the immunosuppressive effects of the tumor, effective DC-based cancer vaccination requires successful TAA presentation and co-stimulatory molecules [194,201]. For this reason, the increased immunogenicity of DC vaccines has led to the use of cytokine combinations and specific DC subsets. Second-generation DC vaccines were created to boost immunogenicity by activating specific DC subsets using cytokine combinations and substances obtained from pathogens [202,203]. For example, TAAs from melanoma-associated antigens, Wilms tumor 1 (WT1), New York esophageal squamous cell carcinoma 1 (NY-ESO-1), and even entire tumor cell extracts can be included in these DC-based vaccines.

To stimulate a more robust immune response, the third generation of DC vaccines leverages blood-derived DCs. The use of DCs derived directly from the bloodstream represents a significant advancement in the rapidly developing field of immunotherapy, eliciting a more robust immunological response than those generated in vitro. One example of this type of DC-based vaccine is the activation of human pDCs with TAA peptides, which significantly enhances CD4^+^/CD8^+^ T-cell responses to specific antigens and produces a quantifiable IFN signature in patients with metastatic melanoma [204]. Likewise, the administration of autologous CD1c^+^ DCs has been associated with prolonged PFS of melanoma patients [205,206].

In summary, DC-based hTERT-targeting vaccines offer novel avenues and are superior to existing options. First, the concept behind DC-based hTERT immunotherapy is straightforward: autologous DCs are cultured in vitro and then reinjected into the human body after they are loaded with the hTERT antigen epitope, thereby generating specific anticancer immune responses [8]. DC-based hTERT-targeted immunotherapy is characterized by high specificity, primarily due to the high affinity of the telomerase antigen epitope for the T cell receptor (TCR), as well as minimal side effects [8]. In line with this, a memory immune response can be developed, making the T cells more effective [207]. As a result, DC vaccines can eliminate tumor cells’ activation of T helper and T cytotoxic cells.

### 5.1. GRNVAC1: Main Dendritic Cell (DC)-Based Vaccine

GRNVAC1 (also known as AST-VAC1) is a DC-based cancer immunotherapy vaccine. The administration of immature patients’ autologous DCs transfected with mRNA encoding a chimeric lysosomal-associated membrane protein-1 (LAMP) and the hTERT protein constitutes the basis of the GRNVAC1 vaccine [131]. The amino acid sequence Tyr-Gln-Thr-Ile, found in the cytoplasmic domain of LAMP-1, matches the Tyr-Xaa-Xaa-hydrophobic amino acid motif, causing increased cell membrane permeabilization and the lysosomal degradation of hTERT protein into a few peptides [208]. Following GRNVAC1 administration, DCs present antigenic epitopes from specific regions of the hTERT protein, thereby triggering polyclonal immune responses [208]. In a study of metastatic prostate cancer, patients who acquired mRNA encoding the chimeric LAMP-hTERT protein by DC transfection showed higher levels of CD4^+^ and CD8^+^ immune response activation than those who were transfected with the DC vaccine loaded with the unmodified hTERT protein [209].

In clinical settings, GRNVAC1 is safe and effective and does not cause notable autoimmune reactions [210]. Patients with acute myeloid leukemia (AML) who received GRNVAC1 therapy showed improved disease-free survival and an hTERT-specific immune response with no relative toxic complications [200,211,212]. Like GRNVAC1, GRNVAC2 is a vaccine alternative based on DCs generated from human embryonic stem cells rather than leukapheresis. The GRNVAC2 vaccine is believed to be more suitable than the GRNVAC1 vaccine, as it bypasses the barriers of delivery [152].

GRNVAC1, which uses autologous DCs loaded ex vivo with hTERT mRNA, is a telomerase-based cancer vaccine that can cause a strong T-cell response against tumor cells that express telomerase. Unlike peptide-based vaccines like GV1001, this approach increases immunogenicity and overcomes the challenge of HLA presentation by targeting a broader range of hTERT epitopes across both HLA class I and II molecules [8,31,213].

### 5.2. Combination of Dendritic Cell (DC)-Based Telomerase Vaccine with Checkpoint Inhibitors

DC vaccines teach the immune system to identify and target certain tumor antigens. ICIs restore T cell function, reducing dysfunctional T cells and preventing immune evasion mechanisms. When these treatments are combined, T-cell activation is improved, reducing the number of exhausted T cells [214,215].

The development of exhausted PD-1^+^ cytotoxic T cells after immunization with a DC-based GRNVAC1 telomerase vaccine has been recognized as a significant obstacle that may hinder the long-lasting anti-tumor efficacy of the GRNVAC1 telomerase vaccine. For this reason, the therapeutic benefit of the GRNVAC1 telomerase vaccine can be enhanced by combining it with other immunotherapeutic options. In particular, combining GRNVAC1 with ICIs (such as anti-PD-1 drugs) and immunomodulatory treatments targeting regulatory T cells and COX2-mediated immunosuppression are approaches to enhance the effectiveness of the GRNVAC1 telomerase vaccine [216].

Although there have been a few Phase II/III clinical studies, some recent studies support combining GRNVAC1, a DC-based telomerase vaccine, with immune checkpoint inhibitors to fight immunosuppressive TME and T-cell exhaustion [217,218,219,220]. Phase I clinical studies combining the GRNVAC1 telomerase vaccine with pembrolizumab demonstrated safety and clinical benefit, with objective response rates of over 50% and nearly one-third of patients achieving complete response in advanced melanoma [137,143]. The molecular mechanism of the combination therapy involves activation of CD4^+^ T helper cells and a reduction in PD1^+^ exhausted T cells [216,217]. In summary, the combination of ICIs and GRNVAC1 DC-based telomerase vaccines has shown improved immunogenicity and promising clinical responses. Even though the clinical benefit of GRNVAC1 can be enhanced by coupling this vaccine with ICIs, conclusive proof of GRNVAC1’s long-term effectiveness is needed from ongoing randomized studies [137,143,217,220].

### 5.3. Other Dendritic Cell (DC)-Based Vaccines

The translational application of other DC-based vaccines has been demonstrated in multiple clinical trials across different malignancies. Initially, researchers explored the use of DCs transfected with the hTERT peptide. In half of patients with advanced breast or prostate cancer, vaccination with ex vivo-generated autologous DCs pulsed with the p540 hTERT peptide, combined with keyhole limpet hemocyanin as an adjuvant, induced the increased infiltration of hTERT-specific T cells. However, only 14% of vaccinated patients experienced an increase in tumor-infiltrating CD8^+^ T cells that was associated with partial tumor regression [221].

The immunologic and clinical consequences of immunizing cancer patients with autologous dendritic cells stimulated with both MHC class I and class II hTERT-derived peptides were examined [199]. The DC immunization effectively stimulated peptide-specific cytotoxic CD8^+^ T-cell responses, demonstrating that DCs loaded with hTERT can elicit increased numbers of CD8^+^ T cytotoxic cells capable of recognizing telomerase-expressing tumor cells and CD4^+^ T helper cells [199]. An increase in circulating regulatory T cells (CD4^+^CD25^+^FoxP3^+^) implies a compensatory immunosuppressive mechanism that could limit the intensity or duration of the antitumor response [199]. Although there were a few cases of cancer patients experiencing tumor regression, several patients reported clinical stability, which was linked to higher T-cell responses [199].

Another example of a dendritic cell-based vaccine was reported in a clinical case study in a patient with pancreatic cancer. In this clinical case study, the patient’s DCs were loaded with hTERT mRNA, which elicited a strong immune response. The patient achieved complete clinical remission following surgery and gemcitabine therapy [61]. In a recent Phase I clinical study, immunogenicity and efficacy were examined in DCs pulsed with three different HLA-A2-restricted tumor peptides (hTERT, carcinoembryonic antigen, and survivin, with the use of the TLR3 agonist poly-IC:LC), in patients with advanced pancreatic cancer [222]. These results suggest that DC vaccines pulsed with peptides elicit strong, long-lasting CD8^+^ and CD4^+^ T-cell responses, indicating that DC immunization can at least partially overcome the challenges posed by an immunosuppressive TME [222]. However, the clinical benefit of peptide-pulsed DC vaccines emerged in specific immune responders [222]. As a result, the positive relationship between strong immunogenicity and improved clinical outcomes in pancreatic cancer encourages the development of DC-based vaccines in combination with treatments that modulate the TME and sustain T-cell activity. Similarly, metastatic melanoma patients were transfected with autologous DCs loaded with survivin, hTERT, and p53-derived peptides to assess the clinical efficacy and immunogenicity of this therapeutic approach in a Phase II trial. Simultaneously, the metastatic melanoma patients received IL-2, metronomic cyclophosphamide, and celecoxib (a Cox-2 inhibitor) to enhance the antitumor immune response. The results showed that patients with disease stabilization exhibited an antigen-specific immune response [223].

In glioblastoma, a Phase I/II clinical trial provided convincing evidence that glioblastoma participants who received DCs transfected with hTERT and survivin mRNA, after completing standard post-operative chemo-radiotherapy, had a strong immunological response without severe toxicity or autoimmune complications. Regarding survival, the vast majority of vaccinated patients experienced longer PFS [224]. The theory was that the vaccination of glioblastoma patients with telomerase-loaded DCs that migrated to local lymph nodes as mature-presenting-tumor antigen epitopes on HLA molecules resulted in an antitumoral T-cell response, without any implication that the DCs directly crossed the BBB [224,225]. All the differences among DC-hTERT vaccines are presented in Table 4.

## 6. The Clinical Efficacy of Telomerase DNA Vaccines and Their Combinations

The ability to easily include many genes that encode either full-length or partial tumor antigens is just one of the many benefits that DNA vaccines offer. Additional approaches that have been explored include using influential viral promoters, codon-optimized genes, immunoglobulin sequences, or increasing CpG patterns in the plasmid backbone. Furthermore, DNA vaccines are stable, safe, and easy to produce. Despite these advantages, early research on DNA vaccines showed that DNA is generally not very immunogenic, especially when examining self-antigens. The effectiveness of DNA vaccination has dramatically improved due to recent advances in EGT, or DNA electroporation, such as the use of a series of high-voltage and low-voltage pulses [227].

The mechanism of action of DNA vaccines is based on the ability to transfect mammalian cells with plasmid DNA via intramuscular injection, resulting in stable, long-term humoral and cellular immune responses due to the constitutive expression of TAAs [228,229]. Furthermore, patients tolerate DNA vaccines well and do not experience any severe side effects [230,231].

In a preclinical setting, a synthetic, highly optimized full-length hTERT DNA vaccine was developed. This phTERT vaccine triggered significant, widespread hTERT-specific CD8+ responses in vivo when administered by electroporation [232]. The phTERT vaccine was shown to induce increased generation of T cells expressing CD107a, IFN-γ, and TNF-α, along with strong antigen-specific perforin secretion. In this context, a protective effect was demonstrated in mice with HPV16 cancer [232].

A characteristic example of a DNA vaccine targeting telomerase is INVAC-1, which contains a dormant form of hTERT [227]. The delivery of the INVAC-1 telomerase vaccine has been shown to induce the activity of CD4^+^ T helper cells and memory CD8^+^ T cytotoxic cells, thereby reducing tumor growth in a mouse model of HLA-A2 persistent and recurrent sarcoma [227].

In a clinical setting, the efficacy of the INVAC-1 vaccine has been demonstrated in recent Phase I trials (NCT02327468) and Phase II trials for solid tumors and chronic lymphocytic leukemia (CLL) [233]. In a Phase I clinical trial (NCT02301754), Teixeira et al. used INVAC-1 as the sole treatment agent in a variety of advanced solid tumors [233]. Interestingly, half of the patients showed signs of disease stability, mounting hTERT-specific CD8^+^ and CD4^+^ T cell immune responses [233]. According to the findings, the majority of cancer patients treated with the INVAC-1 telomerase DNA vaccine demonstrated good safety and immunogenicity in patients who relapsed [233].

In a Phase II trial (NCT03265717), INVAC-1’s efficacy in treating patients with Chronic Lymphocytic Leukemia, either alone or in combination with ibrutinib (a Bruton’s tyrosine kinase inhibitor), was evaluated [78].

In addition to the INVAC-1 telomerase vaccine, other hTERT-based DNA vaccines have been developed. For example, the INO-5401 DNA vaccine was generated with the incorporation of hTERT, prostate-specific membrane antigen, and three synthetic plasmids targeting Wilms’ tumor gene-1 [78]. The combination of INO-5401 with INO-9012 and atezolizumab (anti-PD-L1) in advanced urothelial carcinoma (NCT03502785) or with cemiplimab (anti-PD-1) and chemoradiotherapy in glioblastoma (NCT03491683) was evaluated in two Phase I/IIA trials [78].

Despite considerable progress, no DNA telomerase vaccines have been approved for cancer treatment, underscoring the need for ongoing research to enable their clinical application and to improve their specificity and effectiveness. For this reason, ongoing clinical trials are crucial for evaluating the long-term efficacy and safety of these DNA telomerase vaccines across different tumor subtypes.

## 7. Adoptive Cell Transfer

To improve the recruitment of T cells that target tumor antigens, gene-modified T cell therapy has been developed. This approach is based on the adoptive transfer of T cells that have been genetically modified to express TCRs with high specificity for a particular tumor antigen. ACT is a therapeutic option that first involves the isolation of tumor-infiltrating lymphocytes (TILs) from cancer patients, followed by activating, expanding, and functionally identifying them in vitro before reintroducing them to the patient [234,235].

Although using telomerase as a TAA is promising, the existing research on TCR-engineered T cells that can specifically detect telomerase has been restricted to preclinical studies. The functionality of telomerase-specific TCRs has been demonstrated in both CD4^+^ and CD8^+^ T-cell subsets by engineering TCRs on T cells [236,237,238,239,240]. In particular, researchers identified a TCR sequence called Radium-4 after administering the hTERT peptide 611–626 vaccination to a patient with pancreatic cancer. Specifically, T-cells were modified to express the Radium-4 TCR. They were able to effectively eliminate melanoma cells and patient-derived ascites cells that expressed high hTERT levels without affecting healthy cells [241]. In an initial clinical trial, the effectiveness and reliability of radium-4-based T-cell therapy were evaluated in patients with metastatic non-small-cell lung cancer. To explore the therapeutic potential of TCR immunotherapy for solid tumors, this novel approach used mRNA electroporation for transfection with a TCR linked to the broadly expressed MHC class II molecule [242].

Although adoptive TIL therapy targeting telomerase epitopes is considered a promising treatment option, several challenges hinder its clinical effectiveness. First, the efficacy of TILs against telomerase epitopes is influenced by the different immunosuppressive signals (PD-L1, TGF-β, IDO) present in various cancer types. Vaccines targeting telomerase have not typically been successful, most likely because the TME suppresses telomerase-specific T lymphocytes [243]. As a result, TILs may develop tolerance and anergy since telomerase is a “self” antigen. Second, some cancers reduce the number of molecules in the antigen-processing machinery, thereby limiting TIL recognition of hTERT epitopes [244]. Third, increased neoantigen competition may be present in cancers with high TMB, thereby reducing the prevalence of telomerase-specific T cells [244].

These molecular hallmarks of cancer types can help determine whether adoptive TIL therapy targeting telomerase epitopes is effective. Furthermore, TIL therapy faces limitations in accessibility and effectiveness. On one hand, the challenge with TIL therapy lies in collecting TILs with strong anticancer and high proliferative potential. On the other hand, TIL reinfusion and in vitro expansion are labor-intensive and costly [245]. In addition to the above factors, different treatment schemes can affect the efficacy of adoptive TIL therapy targeting telomerase epitopes. For example, chemotherapy and radiation therapy may increase MHC expression and antigen release, potentially enhancing recognition of telomerase epitopes. In line with this, ICIs can increase telomerase-reactive TIL proliferation and persistence [246].

To expand the range of cell engineering in the fight against cancer, researchers have used genetic engineering to enhance TILs or to produce T cells that express specific TCRs. Numerous innovative treatments have emerged, including TCR-T cells, CAR-T cells, and BiTEs [247]. In the development of CAR cells, a single-chain antibody (ScFv) that recognizes a TAA, along with T cell signaling molecules (such as CD3 and CD28), is required to create a chimeric antigen receptor (CAR) in T cells. T cells are transfected with the CAR, enabling the engineered T cells to recognize the TAA to APCs independently of MHC molecules, leading to cancer cell death [248]. These cells then utilize cytokines, the Fas and Fas ligand axis, and the perforin and granzyme axis to induce cancer cell death [249].

BiTEs can both efficiently stimulate T cells and compromise the tumor burden by combining a single-chain antibody against a tumor antigen with a single-chain antibody against a T-cell surface protein to produce bispecific antibody components [250].

Other cell-based strategies targeting hTERT have been explored at the preclinical level. These include DC transfection with an adenovirus containing the hTERT cDNA sequence, fibroblast-derived AAPCs (artificial antigen-presenting cells) cultured with hTERT-encoding cDNA, hTERT-expressing human umbilical vein endothelial cells (HUVECs), and DCs that were infected with a mannan-modified adenovirus encoding hTERT. A characteristic example of adoptive cell therapy is the transfection of human umbilical vein endothelial cells (HUVECs). In particular, Mu et al. investigated the antitumor immunity elicited by hTERT-expressing human umbilical vein endothelial cells (HUVEC-TERTs) since the immortalized human umbilical vein endothelial cells (HUVECs) were transfected with a lentivirus containing hTERT. Their findings demonstrated that HUVEC-TERTs expressing integrin α5, CD31, and vascular endothelial growth factor receptor-2 (VEGFR-2) had strong telomerase activity [251]. Due to their antiangiogenic properties, these HUVECs may be widely used in cancer immunotherapy to induce specific immune responses [251]. Regarding their ability to induce strong antigen-specific immune responses, all these vaccination models showed promising results [232,251,252,253]. In addition to established cell-mediated TERT approaches, circulating anti-hTERT antibodies in cancer patients’ sera may serve as an anticancer strategy [254,255].

## 8. Limitations of Telomerase Vaccines and Optimization Strategies

Even though tremendous progress has been made with cancer vaccines, tumor heterogeneity, immunosuppressive TME, and immunological tolerance mechanisms significantly hinder their effectiveness. First, cancer vaccines can promote the proliferation of tumor subclones with low antigen expression, a process known as immunoediting, thereby facilitating the immune evasion of those subclones [256]. The limited clinical efficacy of cancer vaccines also results from the interaction between these checkpoints and their ligands inside the tumor microenvironment (TME), which hampers the immune system’s ability to combat cancer cells [256]. In the field of telomerase vaccines, most studies have shown that a significant proportion of vaccinated participants exhibit hTERT-specific immune responses, which do not correlate with the patients’ clinical responses. Only a small number of telomerase-vaccinated cancer patients have successfully achieved improvements in their objective clinical outcomes or at least disease stability. Specifically, disease stabilization has been observed to fluctuate from 16% to 83% among telomerase-vaccinated cancer patients, and the objective response rate ranges from 8% to 77% in the same group. For example, in a phase I study using the improved TERT(572Y) peptide, no tumor regression was observed, although 21% of patients experienced disease stability [153]. Overall, clinical trial data indicate that hTERT-based immunotherapies can effectively trigger specific T cell responses in most individuals; however, the overall clinical outcomes are generally mild. Second, telomerase peptide vaccines alone often fail to generate strong, long-lasting memory T-cell responses needed for sustained tumor control. Therefore, multiple doses of telomerase DNA vaccines are necessary to maintain a persistent immune response. Third, the vaccine’s long-term efficacy may be limited by the development of immunological tolerance to the encoded telomerase epitope, requiring efforts to reduce immune tolerance [115]. Many telomerase peptides are weakly immunogenic because they originate from self-antigens, resulting in central and peripheral tolerance that limits the magnitude of cytotoxic T-cell responses. Another key factor contributing to the limited efficacy of telomerase-based vaccines across different cancer patients is that most telomerase peptide vaccines are designed for specific HLA types, eliciting either only cytotoxic responses or both cytotoxic and helper immune responses.

To enhance the effectiveness of hTERT-targeting therapy, several approaches have been adopted to optimize hTERT vaccines. First, hTERT long peptide or multi-epitope cancer vaccines have arisen, expanding the pool of patients who might benefit from immunotherapy by improving HLA allele coverage. The molecular mechanism of these hTERT long peptides or multi-epitope cancer vaccines is based on the expansion of CD8^+^ and CD4^+^ TERT-specific T cells, including multiple immunogenic MHC-I and MHC-II-restricted hTERT epitopes in vaccines. In fact, CD4^+^ T helper cells are crucial to eliciting a strong and durable immune response against cancer cells. In parallel, these hTERT vaccines can hinder immune-escape mechanisms in the tumor-suppressive environment. In addition to eliminating cancer cells, effector immune cells also help mitigate the tumor microenvironment (TME)’s suppressive barriers. At every stage of carcinogenesis, the TME’s non-malignant cells—such as immunosuppressive myeloid cells and regulatory lymphocytes—contribute to cancer progression through tumor-promoting effects. For this reason, ongoing clinical studies are combining ICIs and hTERT immunotherapy to improve the efficacy of hTERT vaccines. In addition, the efficacy of hTERT vaccines has been evaluated in conjunction with chemotherapy or radiation therapy, as these therapies are known to increase antigen release, thereby enhancing cross-presentation.

Furthermore, vaccine effectiveness is enhanced by the inclusion of adjuvants, such as TLR agonists, cytokines, and ICIs, which boost immunological responses and prolong immune activation. For example, cancer vaccines can be combined with ICIs to mount a strong immune response to delay or inhibit tumor growth in the later stages of cancer [257]. While ICIs can counteract inhibitory signaling pathways, allowing for continuous immune activation, TLR agonists, such as CpG oligodeoxynucleotides (CpG-ODNs), stimulate APCs and enhance antigen presentation. In preclinical and clinical studies, these adjuvants have demonstrated promising efficacy, supporting the development of innovative cancer vaccines. Despite challenges in creating effective cancer vaccines, several therapeutic vaccines are being developed and tested in preclinical and clinical studies [258]. In the field of telomerase vaccines, the clinical effectiveness of hTERT vaccines can be improved by using adjuvants like TLR agonists or Montanide [216]. From a molecular perspective, adjuvants help APCs, such as DCs, present weakly immunogenic telomerase epitopes more effectively, thereby boosting CD4^+^ T helper cell activation and supporting a sustained immune response.

In addition, selecting the right platform for cancer vaccines requires carefully weighing a range of variables, including delivery methods. Different delivery methods, including microbe-based (e.g., bacterium-based therapeutic vaccines) [259], virus-based [260], whole cell-based [261], and nucleic acid-based vectors [262], can be used to administer these therapeutic vaccines.

In the context of cancer vaccine optimization, an important issue is identifying suitable neoantigens expressed by cancer cells. The tumor-associated antigens (TAAs) are overexpressed in several malignancies, but they also are expressed in healthy tissues [263]. Due to “central thymic tolerance” in antigen-specific T cells, owing to TAAs’ poor expression in healthy tissues, anti-tumor T cell immune responses are not sufficiently activated [54,264]. For this reason, tumor cells generate neoantigens due to non-synonymous cell variants, such as gene fusions, point mutations, and RNA editing events [265,266,267,268]. In this context, somatic tumor mutation-acquired neoantigens are highly immunogenic and can evade thymus-negative selection and elicit a strong neoantigen-specific T cell response. The identification of neoantigens and the creation of novel molecular epitopes that the immune system recognizes as foreign to trigger a strong immune response against tumor cells are two of the biggest obstacles in developing cancer vaccines [269].

Last but not least, the limited clinical effectiveness of tumor vaccines can be explained by immune-related adverse events (irAEs). Injection site discomfort, headaches, influenza-like symptoms, fever, nausea, diarrhea, rashes, erythema, pruritus, myalgia, and dyspnea are common adverse effects. Although less frequent, serious adverse effects can include pulmonary embolism, immune system problems, and mental health issues. Additional side effects from vaccines and their adjuvants include hyponatremia, elevated liver enzymes, anemia, colitis, and increased creatinine levels. Moreover, vaccine-induced immune responses, particularly T cell responses, may lead to tumor pseudo-progression [270]. As a result, it is essential to closely monitor and manage these immune-related effects to ensure the safety and efficacy of cancer vaccines.

Despite the large number of clinical trials carried out using telomerase-based vaccines the overall therapeutic effect seen so far has been minimal. These vaccines are reliably shown to be highly immunogenic, eliciting both CD4^+^ and CD8^+^ T-cell responses across a variety of cancer types (melanoma, NSCLC, prostate, pancreatic, and hematologic malignancies). Telomerase-based vaccines are also safe and well-tolerated in all studies, with few autoimmune reactions. This positive safety profile strongly encourages more research into combination schemes.

## 9. Conclusions

This review provides a comprehensive overview of therapies harnessing the immune system to eliminate cancer cells. Vaccines have emerged as recent critical therapeutic modalities in the fight against cancer since they generate specific anti-tumor responses with low systemic toxicity, relying on the host’s tumor immunosurveillance mechanisms. Telomerase is considered a promising anticancer immunotherapeutic approach, since cancer cells naturally express telomerase, which can be targeted to eradicate cancer cells without harming healthy cells. This review provides critical clinical insight into not only the use of telomerase vaccines but also their combination with immunotherapy or chemotherapy. Combining telomerase vaccines with ICIs can overcome TME resistance mechanisms, reinvigorating the immune response.

## Figures and Tables

**Table 1:** Potential biomarkers predicting telomerase vaccine efficacy.

Biomarker	Outcome of Telomerase Vaccine	References
T cell infiltration	GX301-treated cancer patients had increased antigen-specific IFNγ-secreting T cells In NSCLC, GV1001-treated cancer patients were long survivors, showing persistent GV1001-specific T cell memory responses (Phase II, I/II trials) In NSCLC, UV1-treated cancer patients had longer overall survival, related to persistent T-cell responses (Phase I/IIa study) In glioblastoma, UCP-Vax increased infiltration of anti-TERT CD4^+^ T-cells, increasing their overall survival (NCT04280848) In HPV^+^ cancers, UCP-Vax and atezolizumab increased T cell infiltration (phase II study).	[79,83,84,85,86]
IFN-γ levels	In NSCLC, GV1001 treated cancer patients presented increased survival due to high IFNγ levels (Phase II, I/II trials)	[84]
Polyfunctional cytokine patterns	UCP-Vax was reported to induce increased generation of polyfunctional CD4^+^ T cells, offering clinical benefit	[87]
Epitope spreading	In lung cancer, GV1001 clinical responders showed intramolecular epitope spreading In glioblastoma, UCP-Vax ameliorated the median overall survival of cancer patients that presented epitope spreading (Phase II study)	[85,88,89]
TCR repertoire	In clinically responding patients with metastatic melanoma, UV1 and ipilimumab increased TCR repertoire diversification (NCT02275416)	[90]
Vaccine-induced antibody response	In advanced lung cancer, UCP-Vax clinically responders were associated with increased antibody response against hTERT target	[87]

Abbreviations: HPV, human papillomavirus, NSCLC, non-small-cell lung cancer, IFN-γ, interferon-gamma, TCR, T cell receptor.

**Table 2:** Characteristics of telomerase peptide vaccines.

Feature	GX301	Vx-001	UV1	GV1001	UCP-Vax
Antigen target	hTERT (telomerase)	hTERT (telomerase)	hTERT (telomerase)	hTERT (telomerase)	hTERT (telomerase) + survivin
Immune responses	Mixed CD4^+^ CD8^+^ immune responses	CD8^+^ immune responses	CD4^+^ immune responses	Mixed CD4^+^ and CD8^+^ immune responses	CD4^+^ immune responses
HLA typing	Broad HLA typing	(HLA)-I-dependent	HLA-independent	HLA-independent	(HLA)-II-dependent
Adjuvant	Montanide + imiquimod	Montanide	GM-CSF	GM-CSF	DPX lipid-based depot system
Examined cancers	Prostate	NSCLC	Melanoma, mesothelioma, lung and prostate cancer	Pancreatic, lung	HPV-positive malignancies, NSCLC, glioblastoma, HCC

Abbreviations: HLA, human leukocyte antigen, NSCLC, non-small-cell lung cancer, hTERT, human telomerase reverse transcriptase, HPV, human papillomavirus, HCC, hepatocellular carcinoma, GM-CSF, granulocyte-macrophage colony-stimulating factor.

**Table 3:** Clinical data of telomerase-based vaccines.

hTERT Vaccine	Cancer Type	Phase	Outcome	References
UV1 telomerase vaccine and pembrolizumab	Advanced melanoma	Phase I	Clinical benefit with induction of CD4^+^ and CD8^+^ T cell responses	[140]
UV1 telomerase vaccine and ipilimumab	Metastatic melanoma	Phase I/IIa	CD4^+^ T cell activation and increased IFN-γ secretion	[141]
UV1 telomerase vaccine and pembrolizumab	Advanced melanoma	Phase I	Increased survival rates and increased immune responses.	[143]
UV1 telomerase vaccine and ipilimumab	Melanoma		Induction of persistent, long-term CD4^+^/CD8^+^ T-cell immune response by increasing the diversification of TCR repertoire.	[90]
UV1 and ipilimumab and nivolumab	Malignant pleural mesothelioma		A strong immune response but no survival benefit in patients with malignant pleural mesothelioma.	[146]
GV1001 at three doses and GM-CSF	Advanced pancreatic cancer (PDAC)	Phase I/II	The intermediate-dose group had longer median survival and stronger immune responses than other groups.	[162]
GV1001 plus gemcitabine/capecitabine	Advanced PDAC, eotaxin-high subgroup	Phase III (TeloVac)	Patients with high eotaxin showed improved median OS and PFS.	[182]
GV1001	Stage III NSCLC	a phase II trial (CTN-2006) and phase I/II trial (CTN-2000)	Immune responders had longer median PFS and OS compared with nonresponders. Immune responders had durable GV1001-specific T-cell memory responses and presented IFNγ(high)/IL-10(low)/IL-4(low) cytokine profiles.	[84]
GV1001 and temozolomide	Advanced stage IV melanoma		Increased GV1001-induced immune responses were associated with longer survival.	[186]
GV1001 plus chemotherapy	Metastatic colorectal cancer		The GV1001 treated cancer patients showed disease control rate at 90.9%.	[187]
UCP-Vax telomerase vaccine and atezolizumab and bevacizumab	hepatocellular carcinoma (HCC)	NCT05528952	The UCP-Vax-treated patients showed clinical benefit and activation of T cells.	[191]
UCP-Vax	Refractory advanced NSCLC	Phase Ib/IIa	The clinical benefit for UCP-Vax-treated cancer patients was associated with increased CD4^+^ T response.	[188]
UCP-Vax and temozolomide	Newly diagnosed glioblastoma	Phase II	UCP-Vax-treated cancer patients showed increased survival, TERT-specific CD4^+^ responses, and epitope spreading.	[89]

Abbreviations: NSCLC, non-small-cell lung cancer, hTERT, human telomerase reverse transcriptase, HPV, human papillomavirus, OS, overall survival, PFS, progression-free survival, PDAC, pancreatic ductal adenocarcinoma, IFN-γ, interferon-gamma, TCR, T cell receptor, GM-CSF, granulocyte-macrophage colony-stimulating factor, HCC, hepatocellular carcinoma.

**Table 4:** Characteristics of clinical findings of DC-based hTERT vaccines.

DC-Based Telomerase Vaccines	Immune Responses	Clinical Outcomes	Safety	Examined Cancers	References
GRNVAC1 (AST-VAC1)	Broader polyclonal responses, high CD4^+^ and CD8^+^ T cell activation	Effective in AML	No notable autoimmune reactions	Acute myeloid leukemia (AML)	[200,211,212]
GRNVAC1 and pembrolizumab	Activating CD4^+^ T helper cells and reducing PD-1^+^ exhausted T cells	Clinical benefit, with ORR >50%		Advanced melanoma	[137,143]
Autologous DCs pulsed with p540 hTERT peptide	Increased infiltration of hTERT-specific T cells in half of patients	Partial tumor regression		Advanced breast or prostate cancer	[221]
Autologous DCs loaded with survivin and hTERT peptides	Increased T-cell responses	About half of patients had disease stability or tumor regression		Metastatic renal cell carcinoma (RCC)	[226]
hTERT mRNA-loaded autologous DCs	Strong immune response	Complete clinical remission		Pancreatic cancer	[61]
Peptide-pulsed DCs with hTERT and CEA and survivin	Strong, long-lasting CD8^+^ and CD4^+^ T-cell responses	Clinical benefit		Advanced pancreatic cancer	[222]
Autologous DCs loaded with survivin and hTERT and p53 peptides and immune-modulators	Antigen-specific immune response	Disease stabilization		Metastatic melanoma	[223]
DCs transfected with hTERT and survivin mRNA	Strong immunological response	Improved PFS	No severe toxicity or autoimmune complications reported	Glioblastoma	[224]

Abbreviations: DCs, dendritic cells; hTERT, human telomerase reverse transcriptase; AML, acute myeloid leukemia; PD-1, programmed death-1; DCs, dendritic cells; ORR, overall response rate; RCC, renal cell carcinoma; CEA, carcinoembryonic antigen; PFS, progression-free survival.

## Data Availability

Not applicable.
